# Autophagy proteins regulate ERK phosphorylation

**DOI:** 10.1038/ncomms3799

**Published:** 2013-11-18

**Authors:** Nuria Martinez-Lopez, Diana Athonvarangkul, Priti Mishall, Srabani Sahu, Rajat Singh

**Affiliations:** 1 Department of Medicine, Albert Einstein College of Medicine, Bronx, New York 10461, USA; 2 Department of Molecular Pharmacology, Albert Einstein College of Medicine, Bronx, New York 10461, USA; 3 Department of Anatomy and Structural Biology, Albert Einstein College of Medicine, Bronx, New York 10461, USA; 4 Institute for Aging Studies, Albert Einstein College of Medicine, Bronx, New York 10461, USA; 5 Diabetes Research Center, Albert Einstein College of Medicine, Bronx, New York 10461, USA

## Abstract

Autophagy is a conserved pathway that maintains cellular quality control. Extracellular signal-regulated kinase (ERK) controls various aspects of cell physiology including proliferation. Multiple signalling cascades, including ERK, have been shown to regulate autophagy, however whether autophagy proteins (ATG) regulate cell signalling is unknown. Here we show that growth factor exposure increases the interaction of ERK cascade components with ATG proteins in the cytosol and nucleus. ERK and its upstream kinase MEK localize to the extra-luminal face of autophagosomes. ERK2 interacts with ATG proteins via its substrate-binding domains. Deleting *Atg7* or *Atg5* or blocking LC3 lipidation or ATG5–ATG12 conjugation decreases ERK phosphorylation. Conversely, increasing LC3-II availability by silencing the cysteine protease ATG4B or acute trehalose exposure increases ERK phosphorylation. Decreased ERK phosphorylation in *Atg5*^*−/−*^ cells does not occur from overactive phosphatases. Our findings thus reveal an unconventional function of ATG proteins as cellular scaffolds in the regulation of ERK phosphorylation.

## Abstract (web summary)

The spatial organisation of mitogen-activated protein kinase (MAPK) signalling by scaffold proteins is an important determinant of signalling specificity. Martinez-Lopez *et al*. show that pre-autophagosomal structures can also act as scaffolds, recruiting the MAPK ERK1/2 and regulating its phosphorylation.

## Graphical abstract

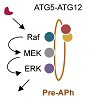

Extracellular signal-regulated kinase (ERK1/2)^1^,^2^ promotes cellular proliferation in response to growth factors. ERK signalling also regulates expression of autophagy and lysosomal genes^3^, while ERK8 has recently been shown to stimulate autophagy by interacting with LC3 (ref. 4). Mice knocked out for p62, an adaptor protein that labels cytoplasmic cargo for autophagic degradation^5^, display ERK hyperphosphorylation^6^, suggesting bidirectional crosstalk between ERK and autophagy. Whether autophagy components regulate ERK1/2 phosphorylation is largely unknown. ERK signalling and autophagosome (APh) formation initiate at the plasma membrane^7^; consequently, we envisioned functional associations between components of the ERK signalling cascade and APh. As the spatio-temporal regulation of ERK activity requires scaffold proteins^1^,^2^,^8^, we hypothesized that APh/LC3-II-positive structures serve as scaffolds or cellular platforms regulating ERK phosphorylation. LC3-II has been shown to be nuclear-localized^9^, however, the function of nuclear LC3 is unclear. Consequently, we investigated whether one of the possible functions of nuclear LC3-II is modulation of nuclear ERK phosphorylation. Recent studies have demonstrated new unconventional functions of autophagy (ATG) proteins in cellular protein secretion^10^,^11^; however, whether ATG proteins or APh regulate cell signalling independent of their role in cargo degradation is largely unknown.

Here we show that ERK cascade components display increased association with ATG5–ATG12-positive pre-autophagosomal structures and with lipidated ATG8 family proteins, LC3-II and GABARAP^12^ following growth factor stimulation. MEK, the upstream ERK kinase, and ERK localize to the extra-luminal face of APh. Inhibition of LC3-II formation in *Atg7*^*−/−*^ liver and brown adipose tissues, *Atg5*^*−/−*^mouse embryonic fibroblasts (MEFs), cells silenced for ATG7, ATG5 or Beclin1, and in a LC3 lipidation-defective mutant decreases cellular and nuclear ERK phosphorylation and activity. We further show that ERK2 colocalizes with ATG5–ATG12, LC3-II and WIPI1 through its substrate-binding domains, and that ATG5–ATG12 conjugation is required for ERK1/2 phosphorylation. Conversely, increasing steady-state LC3-II content by blocking ATG4B-mediated LC3-II delipidation^13^ or acutely exposing cells to the autophagy activator trehalose^14^ increases phosphorylated (P)-ERK levels. Our findings suggest that an unconventional function of the ATG7/ATG5–ATG12/LC3-II cascade regulates ERK1/2 phosphorylation independent of their canonical role in lysosomal proteolysis.

## Results

### ERK cascade components colocalize with ATGs

**Figure 1:**
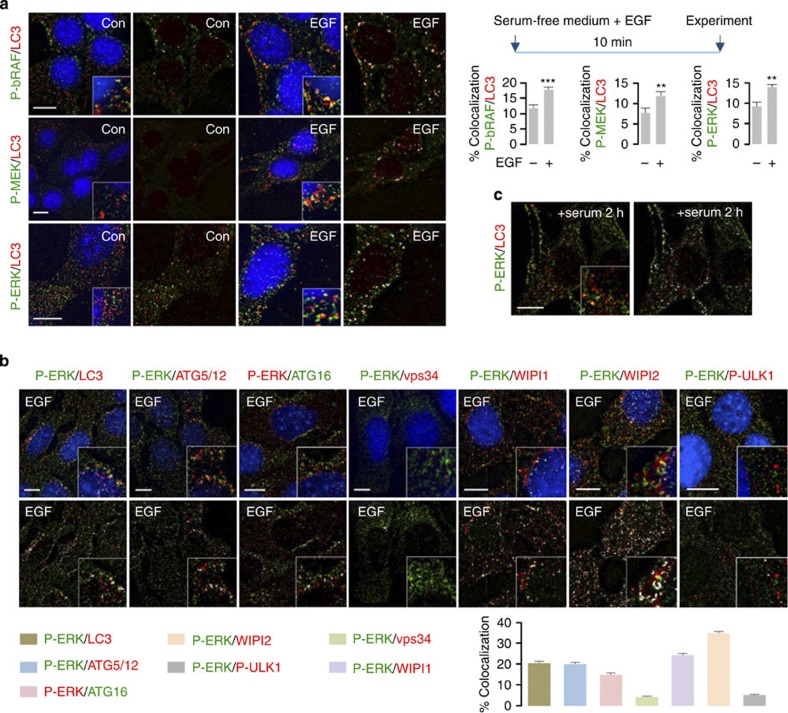
Components of the ERK signalling cascade associate with APh. (**a**) EGF enhances colocalization of phosphorylated (P)-bRAF, P-MEK and P-ERK with LC3. Immunofluorescence (IF) showing colocalization of P-bRAF (green), P-MEK (green) and P-ERK (green) with LC3 (red) in NIH/3T3 cells in presence or absence of EGF (10 min as shown in scheme). The bars represent mean±s.e.m. **P<0.01, ***P<0.001; Student’s *t*-test, 60 cells analysed from two experiments. Scale bars, 10 μm. (**b**) P-ERK colocalizes with pre-autophagosomal and autophagosomal structures. IF depicting colocalization of P-ERK (green or red) with LC3 (red), ATG5–ATG12 (red), ATG16 (green), vps34 (red), WIPI1 (red), WIPI2 (red) and P-ULK1 (red) in EGF-treated NIH/3T3 cells. Scale bars, 10 μm. The bars represent mean±s.e.m. 50 cells analysed from two experiments. (**c**) Growth factors in serum maintain P-ERK/LC3 colocalization. IF depicting colocalization of P-ERK (green) with LC3 (red) in 2 h serum-fed NIH/3T3 cells. (**a**–**c**) Native merged images or images with colocalization highlighted in white pixels are shown. Nuclei are blue (DAPI). Scale bar, 10 μm.

To determine whether ATG proteins regulate ERK phosphorylation, we examined the effect of epidermal growth factor (EGF) treatment on interactions between ERK cascade components and APh/LC3-II. Immunofluorescence in NIH/3T3 cells revealed increased colocalization of ERK cascade components, P-bRAF, P-MEK and P-ERK with LC3-II (Fig. 1a) in response to EGF exposure following brief serum deprivation (scheme in Fig. 1a, right). P-cRAF colocalized with LC3-II in untreated or EGF-treated or serum-fed NIH/3T3 cells (Supplementary Fig. S1a). EGF exposure also increased colocalizations of P-ERK with GABARAP and GATE16 (hereafter collectively referred to as GABARAP) (Supplementary Fig. S1b) but only modestly with the class III PI3K component, vps34 (ref. 15) (Supplementary Fig. S1c). Comparative analysis of EGF-treated NIH/3T3 cells verified that P-ERK colocalized predominantly with autophagosomal (LC3-II) and pre-autophagosomal (ATG5–ATG12 and ATG16) structures and with WIPI (WD-repeat protein interacting with phosphoinositides)-1 and -2 (ref. 16) that are effectors of phosphatidylinositol 3-phosphate (PI3P) in APh formation, but colocalized only modestly with vps34 and P-ULK1 (Fig. 1b). Colocalizations of P-ERK with LC3-II (Fig. 1c) and GABARAP (Supplementary Fig. S1b) were also detected in 2 h serum-fed NIH/3T3 cells indicating that serum-derived growth factors maintained P-ERK/LC3-II and P-ERK/GABARAP colocalizations. To verify our findings of EGF-induced interactions of ERK cascade components with ATG proteins, we exposed cells to EGF following 20 h of serum deprivation, which depletes serum-derived growth factors (scheme in Supplementary Fig. S2a). Indeed, treatment of serum-starved cells with EGF increased colocalizations of P-bRAF, P-MEK and P-ERK with LC3-II, and that of P-ERK with WIPI1 and -2 (Supplementary Fig. S2a). Intriguingly, EGF exposure of 10 min or 20 h serum-deprived NIH/3T3 cells also increased colocalization of the ERK scaffold complex protein KSR1 (kinase suppressor of Ras)^17^,^18^ with LC3-II (Supplementary Fig. S2b), suggesting a possible role for LC3-II-positive structures in providing scaffolding support to regulate the MEK/ERK pathway. We validated P-ERK/LC3-II colocalizations by using two species-distinct P-ERK and LC3 antibodies in EGF-treated NIH/3T3 cells (Supplementary Fig. S3a). Specificity of P-ERK signal was further determined by analysing for loss of total and nuclear P-ERK fluorescence in U0126 (ERK phosphorylation inhibitor)-pretreated NIH/3T3 cells exposed to EGF (Supplementary Fig. S3b). Antibody specificities for P-bRAF, P-cRAF, P-MEK, P-ERK and LC3, and those for fluorescent-tagged secondary antibodies were ascertained by lack of signal in primary antibody- (Supplementary Fig. S3c) or secondary antibody-only NIH/3T3 controls (Supplementary Fig. S3d). Antibody specificities for P-MEK, P-ERK, LC3, vps34, WIPI1 and WIPI2 were also determined in primary antibody-only RALA rat hepatocyte controls (Supplementary Fig. S3e).

P-MEK and P-ERK colocalized with LC3-II in hypothalamic cells (Supplementary Fig. S4a), in 10 min or 20 h serum-deprived RALA hepatocytes exposed to EGF (Supplementary Fig. S4b,c), and in 3T3-L1 preadipocytes cultured in adipocyte differentiation medium (Supplementary Fig. S5a) that acutely activates ERK^19^, reflecting universality of these colocalizations in diverse cells. Agreeing with our findings in NIH/3T3 cells, P-ERK modestly colocalized with vps34 in hypothalamic cells and hepatocytes (Supplementary Fig. S4a–c) but displayed remarkable colocalizations with WIPI1 and WIPI2 in hepatocytes (Supplementary Fig. S4c). Prior phosphorylation of ERK was not required for ERK to colocalize with LC3-II because U0126-pretreatment of NIH/3T3 cells did not decrease EGF-induced ERK/LC3-II colocalization (Supplementary Fig. S5b).

### MEK and ERK localize to the cytoplasmic face of APh

**Figure 2:**
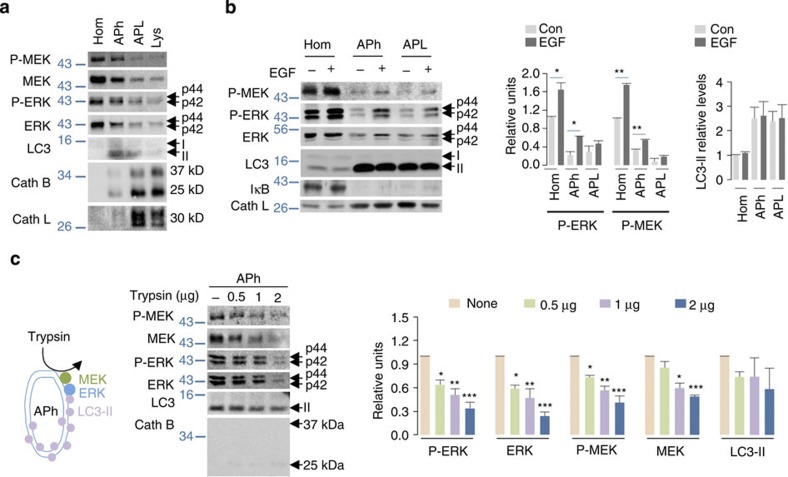
MEK and ERK localize to the cytoplasmic face of APh. (**a**) P- and total MEK and ERK are enriched in APh *in vivo*. Immunoblots for indicated proteins in homogenate (Hom), APh, APL and Lys fractions from livers of fed mice, *n*=4. (**b**) EGF enhances enrichment of P-MEK and ERK in APh fractions *in vitro*. Immunoblots for indicated proteins in Hom, APh and APL fractions from NIH/3T3 cells in presence or absence of EGF (10 min). The bars represent mean±s.e.m. *P<0.05, **P<0.01; Student’s *t*-test, *n*=3. (**c**) P- and total MEK and ERK localize on to the cytoplasmic/extra-luminal face of APh *in vivo*. Left; model depicting localization of MEK and ERK on the extra-luminal face of autophagic vesicles and the ability of trypsin to degrade extra-luminal MEK and ERK, and right; immunoblots for indicated proteins in APh fractions from mice livers untreated (−) or treated with increasing amounts of trypsin for 15 min. The bars represent mean±s.e.m. *P<0.05, **P<0.01, ***P<0.001 compared with corresponding trypsin-untreated value; ANOVA–Bonferroni *post hoc* test, *n*=3. The p44, p42 forms of ERK, LC3-I and membrane-associated LC3-II, and 37 kDa and 25 kDa forms of cathepsin (Cath) B are indicated.

To determine whether MEK and ERK associate with LC3-II-positive autophagic structures *in vivo* we used density-gradient centrifugation to isolate APh, autophagolysosomes (APL), and lysosomes (Lys) from livers of fed mice^20^. Immunoblot analyses of total homogenates (Hom), APh, APL and Lys fractions revealed remarkable enrichment of P- and total MEK and ERK in APh when contrasted to APL or Lys (Fig. 2a). EGF stimulation increased P-MEK and P-ERK levels in APh compared with corresponding fractions from untreated NIH/3T3 cells (Fig. 2b). Increased P-MEK and P-ERK levels in EGF-treated APh (Fig. 2b) were not secondary to cytoplasmic contamination determined by the absence of cytoplasmic protein IκB in APh fractions or due to differences in APh/LC3-II content (Fig. 2b). EGF treatment modestly increased P-MEK and P-ERK levels in APL; however, these increments were not significantly greater than those in APL fractions from untreated cells (Fig. 2b). To distinguish whether MEK and ERK localized at the extra-luminal surface of APh as opposed to sequestration within APh (cartoon depicted in Fig. 2c), we utilized the LC3-II protease protection assay^21^, which is based on the notion that proteins sequestered within APh resist digestion when subjected to controlled protease exposure. Indeed, exposure of APh to increasing amounts of trypsin (0.5, 1 and 2 μg) for 15 min increased the dose-dependent digestion of APh-associated P- and total MEK and ERK and LC3-II levels (Fig. 2c), indicating that MEK and ERK localized to the cytoplasmic surface of APh. Absence of cathepsin B (lysosomal marker) in APh fractions excluded lysosomal contamination of APh fractions (Fig. 2c). Comparative analyses revealed that exposure to trypsin (0.5 μg for 15 min) led to ~50% reduction in APh-associated P-ERK and total ERK levels, and ~25% reduction in APh-associated P-MEK levels, whereas APL and Lys fractions resisted loss of MEK and ERK (Supplementary Fig. S6a). These results indicate two distinct pools of MEK and ERK: one residing on the extra-luminal surface of APh (~50% of total APH-associated ERK), and a sequestered ‘protease-protected’ pool inside APh, APL and Lys most likely destined for degradation. EGF treatment of NIH/3T3 cells did not increase the net flux of LC3-II or p62 to Lys (Supplementary Fig. S6b), suggesting that ERK cascade components interact with APh generated at basal rates.

### ERK cascade components interact with nuclear LC3

**Figure 3:**
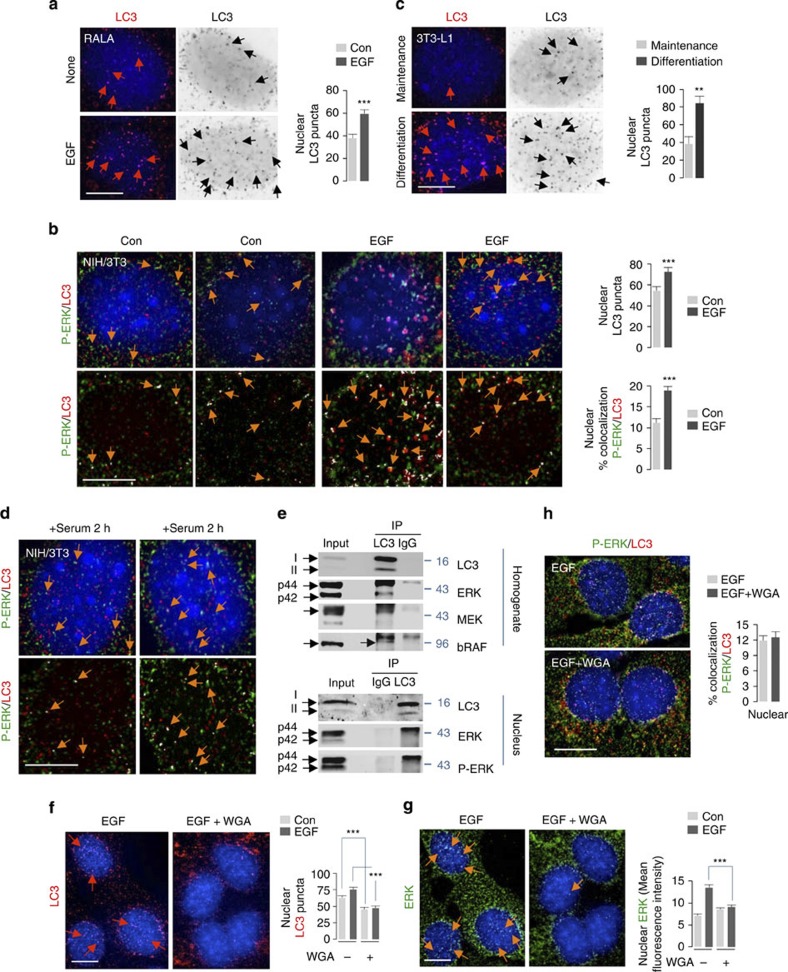
ERK cascade components interact with LC3. (**a**) EGF enhances nuclear LC3-II content in hepatocytes. Immunofluorescence (IF) depicting nuclear LC3-II in RALA hepatocytes in presence or absence of EGF (10 min). Native/inverted images are shown. Scale bar, 5 μm. Bars represent mean±s.e.m. ***P<0.001 compared with control (Con); Student’s *t*-test, 50 cells from two experiments. (**b**) EGF enhances nuclear P-ERK/LC3-II colocalization. IF depicting P-ERK (green)/LC3-II (red) colocalization in untreated (Con) or EGF-treated NIH/3T3 cells. Native images (top)/images with colocalization in white pixels (bottom) are shown. Scale bar, 5 μm. Bars represent mean±s.e.m. ***P<0.001 compared with Con; Student’s *t*-test, 50 cells from two experiments. (**c**) Adipogenic differentiation increases nuclear LC3-II. Images depict nuclear LC3-II in 3T3-L1 preadipocytes in maintenance or differentiation medium (2 h). Scale bar, 5 μm. Bars represent mean±s.e.m. **P<0.01 compared with Con; Student’s *t*-test, 50 cells from two experiments. (**d**) Nuclear P-ERK/LC3-II colocalization in serum-fed cells. IF showing P-ERK (green)/LC3 (red) colocalization in 2 h serum-fed NIH/3T3 cells. Native images (top)/images with colocalization in white pixels (bottom) are shown. Scale bar, 5 μm. (**e**) LC3 interacts with ERK *in vivo*. Immunoblots showing co-immunoprecipitation of LC3 with ERK, MEK and bRAF in homogenate (Hom) (**e**, top), and of LC3 with P- and total ERK in nuclear fractions from mouse livers (**e**, bottom). (**f**) Blocking nuclear transport decreases EGF-induced increase in nuclear LC3-II. LC3 IF (red) in EGF-treated NIH/3T3 cells pre-exposed (30 min) or not to WGA. Bars represent mean±s.e.m. ***P<0.001 compared to with; Student’s *t*-test, 60 cells from *n*=3. Scale bar, 10 μm. (**g**) Blocking nuclear transport decreases nuclear ERK content. ERK IF (green) in EGF-treated NIH/3T3 cells pre-exposed (30 min) or not to WGA. Bars represent mean±s.e.m. ***P<0.001 compared with Con; Student’s *t*-test. (**h**) Blocking nuclear transport does not modify P-ERK/LC3-II colocalization. IF depicting nuclear P-ERK (green)/LC3 (red) colocalization in EGF-treated NIH/3T3 cells pre-exposed or not to WGA. For (**g**) and (**h**): Scale bar, 10 μm, bars are mean±s.e.m. 50 cells from *n*=2. Nuclei are blue (DAPI). Arrows indicate LC3 puncta, ERK or colocalization.

As activation of ERK increases its nuclear localization, we next investigated whether exposure to EGF enhanced P-ERK/LC3-II colocalization in the nucleus. EGF-treated RALA hepatocytes (Fig. 3a) and NIH/3T3 cells (Fig. 3b), and 3T3-L1 preadipocytes exposed to an adipogenic medium (Fig. 3c) displayed increased nuclear LC3-II puncta. EGF exposure also increased P-ERK/LC3-II colocalization in the nucleus compared with untreated NIH/3T3 cells (Fig. 3b). Nuclear P-ERK/LC3-II colocalization was also detected in serum-fed NIH/3T3 cells (Fig. 3d). To verify interactions between ERK and LC3, we co-immunoprecipitated LC3 from total Hom and nuclear fractions from fed mice livers and analysed pull-downs for ERK cascade components. Indeed, analyses of Hom and nuclear fractions revealed that LC3 co-immunoprecipitated with ERK1/2 (Fig. 3e). In addition, bRAF and MEK1/2 were also detected in pull-downs of LC3 from homogenates, indicating interactions of ERK cascade components with LC3.

**Figure 4:**
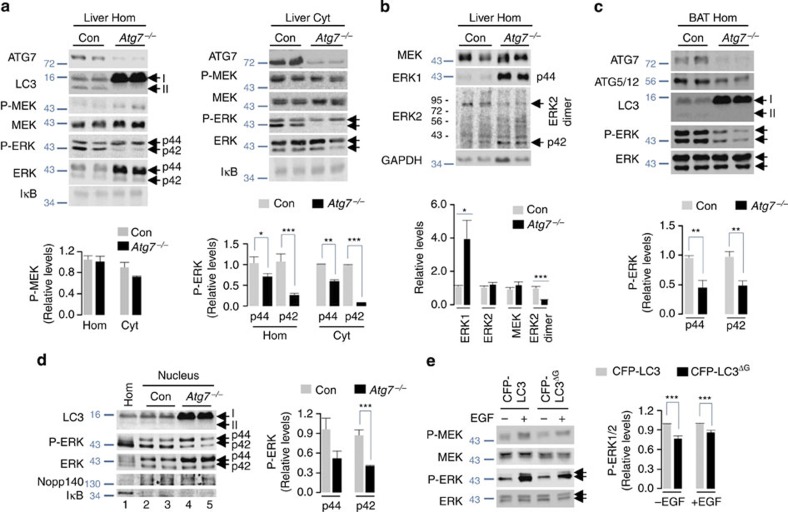
ATG7/LC3-II regulates ERK phosphorylation. (**a**) *Atg7*^*−/−*^ livers display decreased ERK phosphorylation. Immunoblots for the indicated proteins in liver homogenate (Hom) and cytosolic (Cyt) fractions of control (Con) and liver-specific *Atg7*^*−/−*^ mice are shown. The bars represent mean±s.e.m. *P<0.05, **P<0.01, ***P<0.001 compared with Con; Student’s *t*-test, *n*=4. (**b**) *Atg7*^*−/−*^ livers display decreased ERK2 dimers. Immunoblots show the indicated proteins in Hom from Con and *Atg7*^*−/−*^ livers. The bars represent mean±s.e.m. *P<0.05, ***P<0.001 compared to Con; Student’s *t*-test, *n*=4–5. Arrows indicate p42 monomers and dimers. (**c**) *Atg7*^*−/−*^ brown adipose tissues (BAT) display decreased ERK phosphorylation. Immunoblots for indicated proteins in Hom from Con and *Atg7*^*−/−*^BAT. The bars represent mean±s.e.m. **P<0.01 compared with Con; Student’s *t*-test, *n*=4–6. (**d**) *Atg7*^*−/−*^ livers exhibit decreased nuclear ERK phosphorylation. Immunoblots show the indicated proteins in homogenate (Hom, lane 1) and nuclear fractions (lanes 2–5) from Con and *Atg7*^*−/−*^ livers. The bars represent mean±s.e.m. ***P<0.001 compared with Con; Student’s *t*-test, *n*=4–5. (**e**) LC3 C terminus glycine-deleted (ΔG) mutants exhibit decreased ERK phosphorylation. Immunoblots for indicated proteins in total lysates from CFP-LC3- and CFP-LC3^ΔG^-transfected NIH/3T3 cells exposed or not to EGF (10 min). The bars represent mean±s.e.m. ***P<0.001 compared with Con; Student’s *t*-test, *n*=8.

To dissect the dynamics of P-ERK/LC3 interactions in the nucleus, we first investigated whether cytoplasmic LC3-II traverses to the nucleus or whether LC3-II is generated via *de novo* lipidation in the nucleus. To distinguish between these possibilities, we used wheat germ agglutinin (WGA) to block active protein import into the nucleus^22^. WGA pretreatment decreased basal or EGF-induced increase in nuclear LC3-II puncta by ~40–50% (Fig. 3f). The partial reduction in levels of nuclear LC3 puncta in WGA-treated cells (Fig. 3f) and detection of LC3-I in nuclear fractions *in vivo* (Fig. 3e, bottom, and Supplementary Fig. S6c) suggested that *de novo* LC3 lipidation in the nucleus contributes to the total nuclear LC3-II content, although whether a portion of the nuclear LC3 puncta originates from interaction of LC3 with other proteins remains to be elucidated. In support of the possibility that *de novo* LC3 lipidation occurs in the nucleus, nuclear fractions from mouse livers revealed the presence of autophagy proteins required for LC3 lipidation, ATG7, ATG5–ATG12 and ATG16, as well as ATG4B that recycles LC3-I, and the autophagy inducer, ULK1 (Supplementary Fig. S6c). The purity of the nuclear fractions was validated by enrichment of histone3 and absence of cytosolic marker GAPDH or endosomal marker Rab7 or lysosomal marker Cathepsin B (Supplementary Fig. S6c). Moreover, nuclear fractions from *Atg7*^*−/−*^ mouse livers revealed accumulation of soluble LC3-I (Fig. 4d) as typically observed in *Atg7*^*−/−*^ total lysates (Fig. 4a). WGA treatment also decreased nuclear ERK levels in response to EGF (Fig. 3g) as reported^23^; however, WGA treatment did not modify the percentage of P-ERK that colocalized with nuclear LC3-II (Fig. 3h). We next investigated whether ERK gains nuclear entry by ‘piggybacking’ onto LC3-II. LC3-II-deficient *Atg5*^*−/−*^ MEFs did not display reduced nuclear ERK content (Supplementary Fig. S6d), in fact *Atg5*^*−/−*^ MEFs showed increased total ERK levels in the nucleus, indicating that LC3-II *per se* does not traffic ERK into the nucleus.

### Loss of *Atg7* decreases ERK phosphorylation

To analyse the functional consequence of ERK/LC3 interactions on ERK signalling, we subjected total Hom and cytosolic (Cyt) fractions from control and autophagy-deficient *Atg7*^*−/−*^ mouse livers to immunoblot analyses for levels of P- and total bRAF, cRAF, MEK and ERK. Loss of ATG7 did not modify levels of active P-bRAF and P-cRAF (Supplementary Fig. S6e) or P-MEK in Hom and Cyt (Fig. 4a and Supplementary Fig. S13), however, corresponding fractions from *Atg7*^*−/−*^ livers displayed ~50% and ~80–90% reduction in levels of P-ERK1 (p44 isoform) and P-ERK2 (p42 isoform), respectively, when compared with controls (Fig. 4a). Decreases in P-ERK1 and P-ERK2 levels were not secondary to reduced total ERK1 and ERK2 levels; in fact, loss of ATG7 significantly increased total ERK1 levels (Fig. 4a,b) that occurred in part from increased *ERK1* expression (Supplementary Fig. S6f). Fresh *Atg7*^*−/−*^ liver explants also displayed reduced ERK phosphorylation upon exposure to EGF, whereas basal or EGF-induced increases in STAT3 phosphorylation remained intact (Supplementary Fig. S7a), indicating that loss of autophagy did not lead to a global reduction of growth factor signalling. In support of this notion, a second stimulus, menadione, a superoxide radical generator that typically induces ERK phosphorylation^24^, failed to increase P-ERK levels in *Atg7*^*−/−*^ liver explants (Supplementary Fig. S7b) and *Atg5*^*−/−*^ MEFs (Supplementary Fig. S7c). Moreover, reduced ERK phosphorylation occurred independently of any changes in JNK signalling (Supplementary Fig. S7c), as the JNK kinase inhibitor SP600125 (ref. 25) reduced menadione-stimulated c-jun phosphorylation without modifying P-ERK levels in WT cells or rescuing ERK phosphorylation in *Atg5*^*−/−*^ cells. Decreased ERK1 and ERK2 phosphorylation were also detected in *Atg7*^*−/−*^ brown adipose tissues (Fig. 4c), indicating that ATG7 regulates ERK phosphorylation in multiple tissues. Cytoplasmic ERK2 activity requires ERK2 dimerization that in turn requires ERK2 phosphorylation^26^, and agreeing with this notion decreased ERK phosphorylation in *Atg7*^*−/−*^ livers associated with significantly reduced ERK2 dimer levels (Fig. 4b). *Atg7*^*−/−*^ livers also displayed reduced nuclear P-ERK1 and P-ERK2 levels when normalized to corresponding total nuclear ERK levels, independent of changes in nuclear protein loading (equivalent Nopp140 levels) or cytoplasmic contamination (absence of IκB) (Fig. 4d). Decreased ERK phosphorylation in *Atg7*^*−/−*^ livers did not occur due to early loss of autophagy during development because transiently silencing ATG7, ATG5 or Beclin1 in NIH/3T3 cells decreased EGF-induced phosphorylation of ERK2 by ~28, ~33 and ~43% respectively (Supplementary Fig. S7d). In addition, ~44% reduction in ERK1 phosphorylation was observed in NIH/3T3 cells silenced for Beclin1 (Supplementary Fig. S7d).

### Blocking LC3 lipidation decreases ERK phosphorylation

To determine whether ERK phosphorylation requires LC3-II-positive vesicles and not autonomous functions of ATG7 or Beclin1, we transfected NIH/3T3 cells with wild-type (WT) cyan fluorescent protein (CFP)-LC3 or the lipidation-defective C-terminal glycine-deleted (CFP-LC3^ΔG^) construct that impairs APh formation^27^. Equivalent transfection was determined by examining for CFP fluorescence (Supplementary Fig. S7e, left) and CFP-LC3 protein levels in CFP-LC3 and CFP-LC3^ΔG^-transfected cells (Supplementary Fig. S7e, right)^27^. Decreased CFP-LC3 puncta verified the previously described reduction of APh content in CFP-LC3^ΔG^ mutants compared with controls^27^ (Supplementary Fig. S7e, left). As expected, untreated or EGF-treated CFP-LC3^ΔG^ mutants displayed modest albeit significant reduction in ERK phosphorylation independent of changes in MEK phosphorylation (Fig. 4e). Modest reduction (~20–25%) in P-ERK levels in CFP-LC3^ΔG^ mutants suggested that an intact ATG8-lipidation system (E1-, E2- and E3-like ligases) in CFP-LC3^ΔG^ cells likely enabled production of ATG5–ATG12-positive pre-autophagosomal structures or GABARAP-positive vesicles^12^ that allowed residual ERK phosphorylation.

### ERK activity in *Atg5*^*−/−*^ cells depends on nutrients

**Figure 5:**
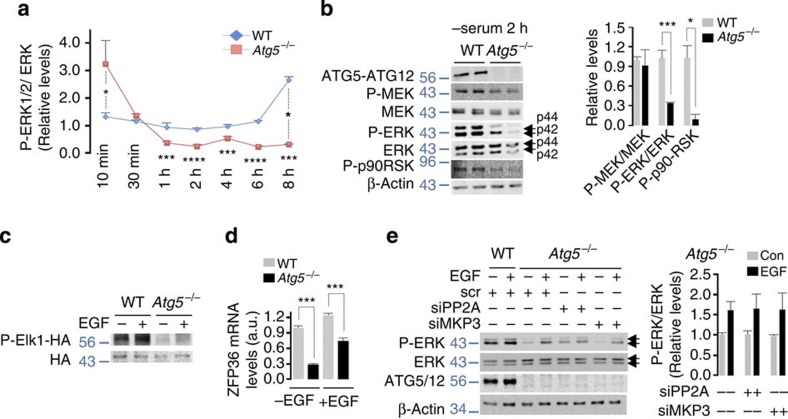
ERK phosphorylation in *Atg5*^−/−^cells depends on nutrient availability. (**a**) *Atg5*^*−/−*^ MEFs display reduced ERK phosphorylation during nutrient deprivation. Quantification of P-ERK levels normalized to total ERK (detected by immunoblotting) in WT and *Atg5*^*−/−*^ MEFs cultured in the absence of serum for indicated times. The bars represent mean±s.e.m. *P<0.05 compared with corresponding WT value; ***P<0.001, ****P<0.0001 compared with 10 min serum-starved *Atg5*^*−/−*^ MEFs; ANOVA-Bonferroni *post hoc* test, *n*=3. (**b**) Serum-deprived *Atg5*^*−/−*^ MEFs display decreased ERK and p90RSK phosphorylation. Immunoblots for the indicated proteins in total lysates from 2 h serum-deprived WT and *Atg5*^*−/−*^ MEFs. The bars represent mean±s.e.m. *P<0.05, ***P<0.001 compared with Con; Student’s *t*-test, *n*=3. (**c**) *Atg5*^*−/−*^ MEFs display decreased nuclear ERK activity. Immunoblots for phosphorylated HA (haemagglutinin)-tagged Elk1 in total lysates from 2 h serum-deprived WT and *Atg5*^*−/−*^ MEFs cells exposed or not to EGF (10 min). (**d**) *Atg5*^*−/−*^ MEFs display decreased Elk1-driven gene expression. Elk1-driven ZFP36 mRNA levels from 2 h serum-deprived WT and *Atg5*^*−/−*^ MEFs cells exposed or not to EGF (10 min). The bars represent mean±s.e.m. ***P<0.001 compared with Con; Student’s *t*-test, *n*=3. (**e**) Decreased ERK phosphorylation in *Atg5*^*−/−*^ MEFs occurs independently of changes in ERK phosphatases. Immunoblots for the indicated proteins in 2 h serum-deprived WT MEFs transfected with scrambled siRNA (scr), and *Atg5*^*−/−*^ MEFs transfected with scr or siRNAs against MKP3 or PP2A in the presence or absence of EGF. The bars represent mean±s.e.m., *n*=3.

To dissect the role of ATG7/ATG5–ATG12/LC3-II cascade in ERK phosphorylation, we analysed P-MEK and P-ERK levels in serum-fed or serum-deprived WT and *Atg5*^*−/−*^ MEFs, both of which display equivalent ATG7 (Supplementary Fig. S7f) and P (Ser555)- and total ULK1 levels (Supplementary Fig. S7g). In consistency with reduced ERK phosphorylation in *Atg7*^*−/−*^tissues (Fig. 4a–d) and in NIH/3T3 cells silenced for ATG7, ATG5 or Beclin1 (Supplementary Fig. S7d), P-ERK levels in *Atg5*^*−/−*^ MEFs rapidly plummeted by 1 h of serum deprivation and remained lower than those in WT MEFs until the end of the experiment at 8 h (Fig. 5a). Serum-deprived *Atg5*^*−/−*^ MEFs displayed decreased phosphorylation of p90 ribosomal S6 kinase (p90RSK), a downstream ERK substrate (Fig. 5b), and decreased levels of phosphorylated Elk1 (nuclear ERK substrate) (Fig. 5c) as well as decreased basal and EGF-induced Elk1-driven *ZFP36* gene expression^28^ (Fig. 5d). We next verified that decreased P-ERK levels in serum-starved *Atg5*^*−/−*^MEFs were not due to overactive ERK phosphatases, MKP3 or PP2A, because transiently silencing MKP3 or PP2A in *Atg5*^*−/−*^ MEFs (Supplementary Fig. S7h) failed to restore ERK phosphorylation in untreated or EGF-treated cells (Fig. 5e). Decreased ERK activity in serum-starved *Atg5*^*−/−*^MEFs did not correlate with increased nuclear localization of transcription factor EB (TFEB) (Supplementary Fig. S8a, left)^3^. In fact, 6 h serum-starved *Atg5*^*−/−*^MEFs displayed decreased nuclear TFEB levels (Supplementary Fig. S8a, left), decreased P-ERK levels (Supplementary Fig. S7f) and reduced TFEB-driven *LAMP-1* and *LC3B* gene expression compared with 6 h serum-starved WT MEFs (Supplementary Fig. S8a, right). These results suggest that other kinases in addition to ERK regulate nuclear availability of TFEB, for instance P-mTOR, a recently elucidated regulator of TFEB^29^, remained unaffected in *Atg5*^*−/−*^MEFs (Supplementary Fig. S7f). In consistency with this idea, rapamycin treatment inhibited mTOR signalling (Supplementary Fig. S8b), and partially restored nuclear TFEB translocation in 6 h serum-starved *Atg5*^*−/−*^MEFs (Supplementary Fig. S8c), without rescuing ERK phosphorylation in *Atg5*^*−/−*^ cells (Supplementary Fig. S8b).

In clear contrast to serum-deprived *Atg5*^*−/−*^ cells, *Atg5*^*−/−*^ MEFs subjected to brief serum deprivation (10 min) displayed raised P-MEK, P-ERK and P-p90RSK levels that increased further with EGF treatment (Supplementary Fig. S8d). We speculated that although *Atg5*^*−/−*^ MEFs do not generate LC3-II (Supplementary Fig. S8d), intact ATG7 in *Atg5*^*−/−*^ MEFs allowed production of vesicles positive for GABARAP^12^,^30^, a mammalian homologue of yeast ATG8, which facilitated ERK phosphorylation in absence of LC3-II. Consequently, we first examined GABARAP levels in *Atg5*^*−/−*^ MEFs. In contrast to *Atg7*^*−/−*^ livers that exhibit reduced GABARAP-II levels (Supplementary Fig. S8e), serum-fed *Atg5*^*−/−*^ MEFs displayed increased GABARAP puncta compared with WT MEFs (Supplementary Fig. S9a). In fact, GABARAP content in serum-fed *Atg5*^*−/−*^ MEFs were comparable to levels detected in 2 h serum-deprived WT MEFs (Supplementary Fig. S9a), indicating that *Atg5*^*−/−*^ MEFs generated GABARAP-positive vesicles possibly from preserved ATG7 and redundant E3-like activity. Most notably, serum-fed (Supplementary Fig. S9b) or EGF-treated *Atg5*^*−/−*^cells (Supplementary Fig. S9c) displayed increased colocalization of P-ERK with GABARAP puncta. To determine whether ATG7 E1-like activity regulated ERK phosphorylation in serum-fed *Atg5*^*−/−*^MEFs, we transiently silenced ATG7 in *Atg5*^*−/−*^cells and examined the effect of loss of ATG7 on MEK and ERK phosphorylation. Although silencing ATG7 did not consistently decrease ERK phosphorylation, possibly from residual ATG7 activity, partial loss of ATG7 significantly decreased basal and EGF-induced MEK phosphorylation in *Atg5*^*−/−*^cells (Supplementary Fig. S9d). In totality, these findings in *Atg7*^*−/−*^ tissues, *Atg5*^*−/−*^ MEFs, ATG7-, ATG5- or Beclin1-silenced cells, and in a LC3 lipidation-defective mutant support our hypothesis that the ATG7/ATG5–ATG12/LC3-II cascade is required for ERK phosphorylation.

### ERK2 interacts with ATGs via its substrate-binding domains

**Figure 6:**
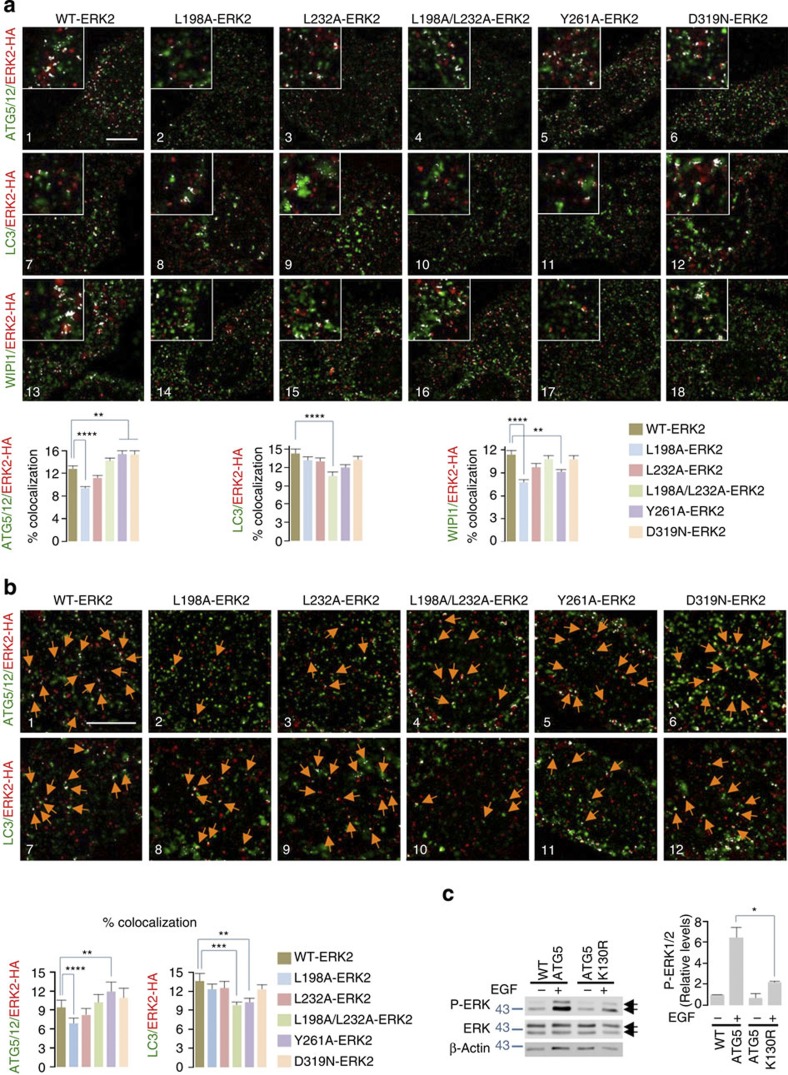
ERK2 utilizes kinase-docking domains to interact with ATG5–ATG12 and LC3-II. (**a**) Mutations in FRS on ERK2 decrease colocalization of ERK2 with ATG5–ATG12, LC3 and WIPI1. Immunofluorescence (IF) showing colocalization (depicted as white pixels by ‘colocalization finder application’) of WT-ERK2-HA, FRS ERK2 mutants (L198A-, L232A-, L198A/L232A-, Y261A-ERK2-HA) or common docking (CD) mutant (D319N-ERK2-HA) with ATG5–ATG12 (panels 1–6), LC3 (panels 7–12) or WIPI1 (panels 13–18) in EGF-treated NIH/3T3 cells. ERK2 is stained in red, and autophagy proteins are stained in green. Scale bar, 10 μm. The bars represent mean±s.e.m. **P<0.01, ****P<0.0001 compared with WT-ERK2-transfected cells; Student’s *t*-test, 50 cells analysed from *n*=2. (**b**) Mutations in FRS on ERK2 decrease colocalization of ERK2 with nuclear ATG5–ATG12 or LC3. IF showing colocalization (white pixels) of WT-ERK2-HA (red), FRS ERK2 mutants (L198A-, L232A-, L198A/L232A-, Y261A-ERK2-HA) or common docking (CD) mutant (D319N-ERK2-HA) with ATG5–ATG12 (panels 1–6) or LC3-II (panels 7–12) in EGF-treated NIH/3T3 cells. Scale bar, 5 μm. The bars represent mean±s.e.m. **P<0.01, ***P<0.001, ****P<0.0001 compared with WT-ERK2-transfected cells; Student’s *t*-test, 50 cells analysed from *n*=2. Arrows depict colocalization. (**c**) ATG5–ATG12 conjugation is required for ERK phosphorylation. Immunoblots for P-ERK, total ERK and β-actin in NIH/3T3 cells transfected with WT ATG5 or the conjugation-defective ATG5 K130R mutant and treated with EGF (10 min). The bars represent mean±s.e.m. *P<0.05 compared with WT ATG5-transfected cells; Student’s *t*-test, *n*=3.

As distinct kinase-docking domains on ERK2 allow its interaction with substrates, we investigated whether ERK2 utilized these substrate-binding domains to interact with ATG proteins. ERK2 interacts with its substrates via acidic residues in its C terminus (Asp316 and Asp319, termed common docking (CD) domain) that coordinate with Thr157/158 (ED domain) to modulate CD domain-substrate interaction. ERK2 also utilizes its F-site recruitment sites (FRS) that include residues Leu198, Tyr231, Leu232, Leu235 and Tyr261 (ref. 31) to interact with substrates. Intriguingly, ERK2 FRS (L198A/L235A) or CD/ED (T157A/D319N) mutants have been reported to exhibit ~40% reduction in MEK1-mediated phosphorylation with the greatest degree of ERK2 phosphorylation inhibition (~70%) in combined ED/FRS (T157A/L198A/L235A) mutants (ref. 31), leading to the speculation that in all likelihood these mutations compromise MEK1–ERK interaction^31^. Consequently, we sought to determine whether ERK2 utilized FRS or CD substrate-binding domains to interact with ATG proteins, and that decreased interactions of these mutants with ATG proteins contributed, at least in part, to the reported decrease in phosphorylation of these ERK2 mutants^31^. To test this possibility, we examined the effect of mutations in FRS (L198A, L232A, L198A/L232A and Y261A)^32^ and CD (D319N)^32^ domains on colocalization of ERK2 with endogenous ATG5–ATG12, LC3-II, WIPI1 and WIPI2 in EGF-treated NIH/3T3 cells. Indeed, while L198A-ERK2 mutants displayed decreased colocalizations with ATG5–ATG12 and WIPI1 (Fig. 6a), mutations at both Leu198 and Leu232 were required to significantly decrease colocalization of ERK2 with LC3-II (Fig. 6a). In contrast, Y261A-ERK2 mutants displayed modestly decreased colocalization with WIPI1, but increased colocalizations with ATG5–ATG12 (Fig. 6a) and WIPI2 (Supplementary Fig. S10a). Mutations at Leu198 and/or Leu232 did not modify ERK2/WIPI2 colocalization (Supplementary Fig. S10a). ERK2 FRS domain (L198A or L198A/L232A) mutants also displayed decreased colocalization with ATG5–ATG12 or LC3-II in the nuclear compartment (Fig. 6b). Decreased interactions of ERK2 FRS domain mutants with ATG5–ATG12, LC3-II and WIPI1 correlated with reduced ERK2 phosphorylation reported in these substrate-binding mutants^31^.

Conversely, ERK substrates interact with ERK2 through substrate D-domains that include basic residues followed by a hydrophobic LXL motif or via substrate F-sites that consist of the FXFP motif^33^,^34^. Analyses of ATG5, ATG12, WIPI1, WIPI2, LC3B and GABARAP sequences for FXFP and LXL domains revealed the presence of the bonafide LXL motif only in ATG12 and LC3B (Supplementary Fig. S10b), and absence of these domains in other ATG proteins examined (Supplementary Fig. S10b). The absence of FXFP or LXL domains in ATG5, and the presence of the LXL domain in ATG12 suggest that P-ERK’s interaction with ATG5–ATG12 conjugate (Fig. 1b) occurs in all likelihood through ATG12 and not via ATG5. Consequently, to determine the role of ATG5–ATG12 conjugation in the regulation of ERK phosphorylation, we examined for levels of EGF-driven ERK1/2 phosphorylation in the conjugation-defective ATG5 K130R mutant^35^, which fails to bind to ATG12. Indeed, EGF-treated NIH/3T3 cells transfected with the ATG5 K130R mutant displayed decreased ERK phosphorylation compared with WT ATG5-transfected counterparts (Fig. 6c), indicating that activation of autophagy, and consequently, availability of ATG5–ATG12 conjugate regulates the degree of ERK phosphorylation. Our findings cannot exclude the possibility that ERK2 may also interact with ATG proteins lacking bonafide ERK-binding sites through alternate yet-unknown binding sites, as observed with the ERK2 substrate stathmin^31^, which also lacks typical ERK2 binding sites. Overall, these results indicate redundancy in the availability of ATG proteins that could potentially serve as signalling platforms to maintain ERK phosphorylation.

### Depleting ATG4B increases LC3-II and ERK phosphorylation

**Figure 7:**
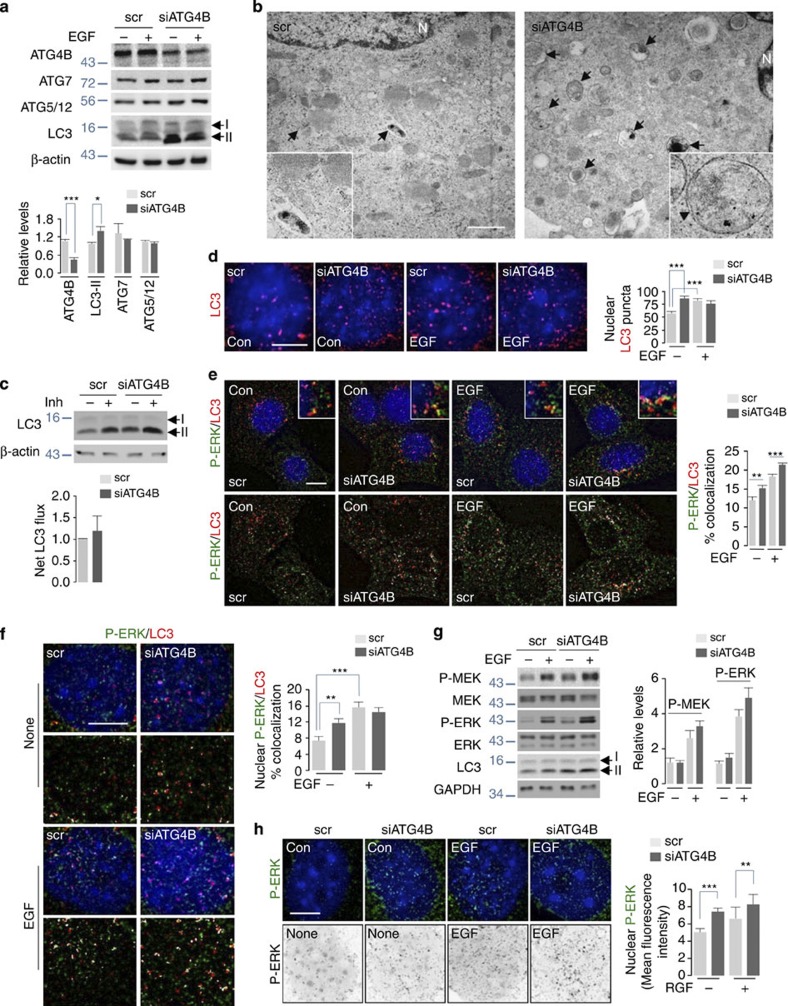
Silencing ATG4B increases LC3-II content and ERK phosphorylation. (**a**) ATG4B depletion increases steady-state LC3-II levels. Immunoblots for indicated proteins in NIH/3T3 cells transfected with scrambled siRNAs (scr) or siRNAs against ATG4B (siATG4B) in presence/absence of EGF. Bars represent mean±s.e.m. *P<0.05, ***P<0.001 compared with scr; Student’s *t*-test, *n*=4. (**b**) siATG4B cells exhibit increased autophagic vesicles. Representative electron micrographs depicting autophagic vesicles (indicated by black arrows) in scr and siATG4B NIH/3T3 cells. (**c**) ATG4B depletion does not increase autophagic flux in serum-fed cells. Immunoblots for indicated proteins in lysates from scr or siATG4B NIH/3T3 cells in presence/absence of lysosomal inhibitors, ammonium chloride and leupeptin (Inh) for 2 h. Scale bars, 1 μm. Bars represent mean±s.e.m., *n*=3. (**d**) siATG4B cells display increased nuclear LC3-II. Images for LC3-II (red) in scr or siATG4B NIH/3T3 cells in presence/absence of EGF (10 min). Scale bar, 5 μm. Bars represent mean±s.e.m. ***P<0.001 compared with scr; Student’s *t*-test, 60 cells from *n*=2. (**e**) ATG4B deficiency increases P-ERK/LC3-II colocalization. Immunofluorescence (IF) for P-ERK (green)/LC3 (red) colocalization in scr or siATG4B NIH/3T3 cells in presence/absence of EGF (10 min). Scale bars, 10 μm. Bars represent mean±s.e.m. **P<0.01, ***P<0.001 compared with scr; Student’s *t*-test, 60 cells from *n*=2. (**f**) ATG4B-deficient cells display increased nuclear P-ERK/LC3 colocalization. IF depicting P-ERK (green)/LC3 (red) colocalization in nuclei of scr or siATG4B NIH/3T3 cells in presence/absence of EGF. Scale bars, 5 μm. Bars represent mean±s.e.m. **P<0.01, ***P<0.001 compared with scr; Student’s *t*-test, 60 cells from *n*=2. (**g**) ATG4B deficiency augments EGF-induced MEK and ERK phosphorylation. Immunoblots for P- and total MEK and ERK, LC3 and GAPDH in total lysates from scr or siATG4B NIH/3T3 cells in presence/absence of EGF. Bars represent mean±s.e.m., *n*=3. (**h**) ATG4B deficiency increases nuclear P-ERK levels. Native (top)/inverted images (bottom) showing nuclear P-ERK content. P-ERK fluorescence in untreated (Con)/EGF-treated scr or siATG4B NIH/3T3 cells is shown. Scale bars, 5 μm. Bars represent mean±s.e.m. **P<0.01, ***P<0.001 compared with scr; Student’s *t*-test, 60 cells from *n*=2. Nuclei are blue (DAPI).

ATG4B modulates LC3-II availability by cleaving LC3 C-terminal glycine and allowing LC3 lipidation^36^, and delipidates LC3-II to recycle LC3-I. We thus investigated whether depleting ATG4B levels modifies LC3-II content, which affects P-ERK/LC3-II interaction and ERK phosphorylation. Partial loss of ATG4B (~50%) in NIH/3T3 cells did not affect ATG7 or ATG5–ATG12 levels, but increased steady-state LC3-II levels (~30%) (Fig. 7a) similar to elevated LC3-II levels in *Atg4B*^*−/−*^heart and brain^37^. We verified increased autophagic vacuolar content in ATG4B-deficient cells by direct visualization of double-membrane vesicular structures containing sequestered cytoplasmic cargo (Fig. 7b), although increased LC3-II content in ATG4B-deficient cells did not translate to increased LC3-II flux in serum-fed conditions (Fig. 7c). ATG4B deficiency also increased nuclear LC3-II to levels comparable to those observed in EGF-treated scrambled siRNA-transfected controls (Fig. 7d). Most notably, EGF treatment increased P-ERK/LC3-II colocalization in ATG4B-deficient cells (Fig. 7e). ATG4B-deficient cells also displayed ~40% increase in nuclear P-ERK/LC3-II colocalization (Fig. 7f), although equivalent P-ERK/LC3-II colocalizations were detected in EGF-treated controls and ATG4B-deficient cells (Fig. 7f). In consistency with our idea that LC3-II is required for ERK phosphorylation, augmenting LC3-II via partial loss of ATG4B increased P-ERK levels in untreated or EGF-treated cells (Fig. 7g and Supplementary Fig. S14). We also observed significantly increased P-ERK levels in the nuclear compartment of ATG4B-deficient cells regardless of EGF exposure (Fig. 7h). In addition, short-term (2 h) exposure of trehalose, which activates autophagy independent of the mTOR pathway^14^, enhanced ERK1/2 phosphorylation (Supplementary Fig. S11a). Enhanced ERK phosphorylation occurred in an autophagy-dependent manner, as trehalose treatment for 2 h failed to augment ERK phosphorylation in *Atg5*^*−/−*^ MEFs (Supplementary Fig. S11a). Although 12 h or 24 h of trehalose treatment robustly increases LC3-II levels compared with 2 h of trehalose treatment (Supplementary Fig. S11b), prolonged trehalose exposure did not increase ERK phosphorylation to levels greater than those observed in untreated controls (Supplementary Fig. S11b). We suspect that robust autophagy activation following prolonged trehalose exposure involves rapid turnover of available LC3-II-positive vesicles, which decreases the availability of LC3-II-positive structures to regulate ERK phosphorylation.

## Discussion

**Figure 8:**
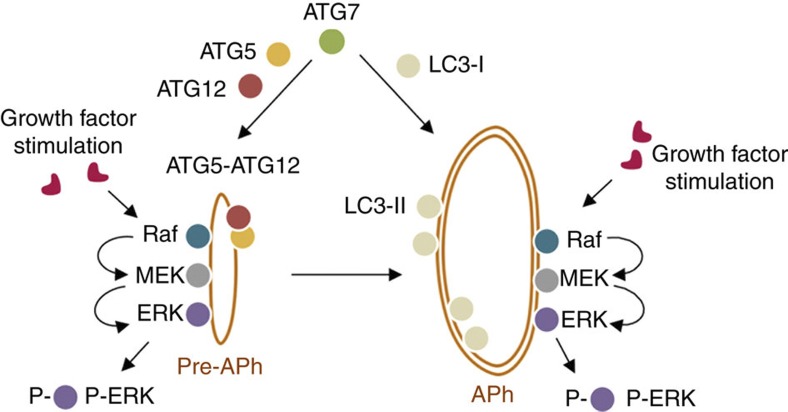
Autophagy proteins regulate ERK phosphorylation. We propose that growth factor stimulation results in the docking of the ERK cascade components, Raf, MEK and ERK onto the cytoplasmic face of autophagic structures. ATG5–ATG12-positive preautophagosomes and LC3-II-positive membranes serve as scaffolds or cellular signalling platforms that facilitate efficient spatial coordination of the Raf–MEK–ERK cascade and thus facilitate growth factor-induced ERK phosphorylation.

In totality, our results demonstrate that the cellular availability of autophagic structures determines the degree of ERK phosphorylation. We speculate that LC3-II-positive membranes and ATG5–ATG12-positive preautophagosomes could serve as scaffolds or cellular signalling platforms that would allow efficient spatial coordination of the Raf-MEK-ERK cascade and thus facilitate ERK phosphorylation (modelled in Fig. 8). In support of this notion, we observe that MEK and ERK localize to the cytoplasmic face of APh, and that ERK2 interacts with these structures through its substrate-binding domains. Furthermore, deleting *Atg7* or blocking LC3 lipidation or ATG5–ATG12 conjugation decreases ERK phosphorylation independent of changes in MEK phosphorylation. We also find that deleting *Atg5* modifies ERK phosphorylation depending on the nutrient status of the cell. While serum starvation of *Atg5*^*−/−*^cells for >1 h reduced ERK phosphorylation and its ability to phosphorylate p90RSK, transiently removing serum for 10 min increased ERK phosphorylation in *Atg5*^*−/−*^cells. *Atg5*^*−/−*^ cells are unable to generate LC3-II and are completely deficient in canonical autophagy; however, surprisingly we observed increased basal GABARAP puncta in *Atg5*^*−/−*^ cells. Thus, it remains possible that during early nutrient deprivation, *Atg5*^*−/−*^ LC3-II-deficient cells utilize GABARAP-positive structures as cellular platforms for ERK phosphorylation, which is supported by our observation of increased GABARAP/P-ERK colocalization in *Atg5*^*−/−*^ cells. These results indicate a functional redundancy in the ability of multiple ATG proteins, that is, lipidated LC3-II, ATG5–ATG12 conjugate or GABARAP-positive vesicles, to regulate ERK phosphorylation. It is thus not surprising that blocking individual ATG proteins, for instance, LC3 lipidation or ATG5–ATG12 conjugation only modestly decreases ERK phosphorylation, whereas deleting the upstream E1-like ligase *Atg7* robustly blocks ERK phosphorylation.

It is reasonable to argue that reduced cargo degradation in autophagy-deficient cells decreases ATP production, and this in turn could have limited ERK phosphorylation; however, intact phosphorylation of JNK, mTOR, STAT3 and ULK1 in *Atg5*^*−/−*^ cells excludes the possibility that ATP depletion has a role in decreased ERK phosphorylation. Overactivation of phosphatases was also excluded as a factor contributing to decreased ERK phosphorylation in *Atg5*^*−/−*^ cells, as silencing *bona fide* ERK phosphatases, MKP3 or PP2A failed to restore ERK signalling in *Atg5*^*−/−*^ cells.

The physiological outcome of changes in ERK phosphorylation in cells with aberrant autophagy remains to be determined. We predict that the previously described increases in cell death in autophagy-deficient cells^38^ or cellular senescence from reduced autophagy with age^39^,^40^ may have occurred, in part, from decreased ERK activity. We also predict that some of the previously reported effects of loss of autophagy on adipocyte mass and differentiation^41^,^42^ may occur in part from impaired ERK-regulated adipocyte differentiation^19^. Hyperactive Raf-MEK-ERK signalling is implicated in hepatocellular cancers, and a recent study has reported increased cell death in Sorafenib (Raf inhibitor)-treated hepatoma cells knocked down for ATG7 (ref. 43), suggesting that therapeutic benefit in Sorafenib-treated ATG7-deficient hepatoma cells may have occurred from a more robust inhibition of ERK signalling. Aberrant autophagy may potentially link ERK hyperphosphorylation to the development of ERK-dependent tumours. In light of these associations, manipulating ATG5–ATG12 and LC3-II availability could be a therapeutic strategy against multiple disorders stemming from altered ERK activity.

## Methods

### Animals and cells

Liver- and BAT-specific *Atg7*^*−/−*^mice were generated by crossing ATG7^F/F^ mice^44^ (a gift from Drs M. Komatsu and K. Tanaka, Tokyo Metropolitan Institute of Medical Science, Japan) with Albumin-Cre or Myf5-Cre mice (Jackson Laboratories), respectively. Studies were performed in 3–6-month-old male mice using a protocol approved by the Institutional Animal Care and Use Committee. Mice were fed a regular chow diet (5058; Lab Diet, St Louis, MO, USA) and maintained on 12 h light/dark cycles. NIH/3T3 cells were cultured in Dulbecco’s Modified Eagle’s Medium (DMEM) (Gibco) supplemented with 10% Newborn Calf Serum (Gibco) and 1% penicillin/streptomycin (P/S) (Gibco). Wild type and *Atg5*^*−/−*^ MEFs (kindly provided by Dr Noboru Mizushima^45^, Tokyo Medical and Dental University, Japan) were cultured in DMEM and 10% FBS. Mouse hypothalamic N41 cells and RALA255-10G rat hepatocyte cells were cultured in DMEM supplemented with 10% and 4% FBS, respectively. 3T3-L1 preadipocytes were differentiated in DMEM supplemented with 160 nM insulin, 250 nM dexamethasone, 0.5 mM 1-methyl-3-isobutylxanthine and 10% FBS^41^. For EGF treatment, cells were washed twice with phosphate-buffered saline (PBS) pre-warmed at 37 °C to deplete serum and then cultured in serum-deprived DMEM supplemented with 1% P/S. Cells were treated with EGF (BD Biosciences) at 25 ng ml^−1^ for 10 min.

### Chemicals and antibodies

Antibodies (Supplementary Table S1) for ATG7, ATG4B, LC3B, P-bRAF (Ser445), bRAF, P-cRAF (Ser338), cRAF, P-p90RSK (Thr359/Ser363), P-MEK1/2 (Ser217/221), MEK1/2, P-ERK1/2 (Thr202/Tyr204) (rabbit and mouse species-specific), ERK1/2, ERK1, ERK2, P-Elk1 (Ser383), P-mTOR (Ser2448), mTOR and P-ULK1 (Ser555) were from Cell Signaling (Danvers, MA, USA); ATG5–ATG12 and ULK1 from Novus Biologicals (Littleton, CO, USA); p62 from Enzo life sciences (Plymouth Meeting, PA, USA); ATG16, Cathepsin B, Cathepsin L, GABARAP and GATE16 (sc-28938), P-c-jun (Ser63/73), c-jun, P-JNK (Thr183/Tyr185), JNK, IϰBα, Rab7 and TFEB were from Santa Cruz Biotechnology (Santa Cruz, CA, USA); Beclin1 from BD Biosciences (San Jose, CA, USA); GAPDH, WIPI1 and WIPI2 antibodies from Abcam (Cambridge, MA, USA); Histone3 from Millipore (Billerica, MA, USA), β-actin from Sigma-Aldrich and JL-8 for CFP detection from Clontech (Mountain View, CA, USA). Nopp140 antibody was a kind gift from Dr U. Thomas Meier (Albert Einstein College of Medicine). Leupeptin (100 μM) and Rapamycin (100 nM) from Fisher Scientific; ammonium chloride (20 mM) from American Bioanalytical; U0126 (10 μM) from Cell Signaling; menadione (50 μM), trypsin and trehalose (100 mM)^14^ from Sigma-Aldrich, WGA (5 μg ml^−1^)^22^ from Invitrogen and SP600125 (ref. 25) (10 μM) from Millipore.

### APh isolation and LC3-II protease protection assay

APh, APL and Lys were isolated from mouse livers and NIH/3T3 cells by differential centrifugation using discontinuous density gradients of metrizamide^20^,^46^,^47^. Briefly, cell or tissue homogenates were centrifuged at 2,000 *g* for 5 min followed by centrifugation of the supernatant at 17,000 g (12 min). Pellets were resuspended in 1.9 ml 0.25 M sucrose and 2.8 ml of metrizamide (85.6%) was added. Samples were centrifuged on a 26–24–20–15% metrizamide gradient at 75,334 g for 3 h. After centrifugation, the APh fraction was collected from the 15–20% interface, APL from the 20–24% interface and Lys from the 24–26% interface. Fractions were centrifuged at 24,000 *g* and pellets were suspended in 0.25 M sucrose and used for assays. For the LC3-II protease protection assay^21^, freshly isolated APh, APL and Lys were incubated in presence or absence of increasing amounts (0.5 μg, 1 μg and 2 μg) of trypsin (Sigma-Aldrich, MO, USA) at room temperature (RT) for 15 min. Samples were quickly boiled in 2 × SDS–PAGE sample buffer at 95 °C for 5 min and resolved by SDS–PAGE. APh, APL and Lys samples were analysed for levels of phosphorylated (P) and total MEK and ERK. Extent of loss of P- or total MEK and ERK in trypsin-treated APh, APL or Lys fractions was expressed as a percentage of total levels in corresponding trypsin-untreated control fractions.

### Subcellular fractionation

For subcellular fractionation, 80% confluent cells or liver pieces (50 mg) were resuspended in buffer (5 mM Na_2_HPO_4_ (pH 7.4), 50 mM NaCl, 150 mM sucrose, 5 mM KCl, 2 mM dithiothreitol, 1 mM MgCl_2_, 0.5 mM CaCl_2_ and 0.2% NP40, supplemented with protease/phosphatase inhibitors), and homogenized using a dounce homogenizer (20 strokes/4 °C), and incubated on ice for 5 min. Cytosolic and nuclear fractions were isolated using ProteoExtract subcellular proteome extraction kit (EMD Millipore, Billerica, MA, USA) as per manufacturer’s instructions. Equivalent protein loading was verified using antibodies for β-actin, glyceraldehyde 3-phosphate dehydrogenase, histone3 or Nopp140.

### Autophagy LC3 flux assays

Flux assays were used to quantify autophagy activity wherein accumulation of autophagy substrates, LC3-II or p62, in the presence of inhibitors of lysosomal proteolysis, ammonium chloride (20 mM) and leupeptin (100 μM), reflects activity. Briefly, cells were cultured in the presence or absence of lysosomal proteases for 2 h following which, cells were collected and lysed and subjected to immunoblotting for LC3-II or p62. Autophagy flux was determined by subtracting the densitometric value of inhibitor-untreated LC3-II or p62 from corresponding inhibitor-treated values.

### LC3 co-immunoprecipitation

Fresh livers were homogenized in 500 μl of 50 mM Tris/HCl (pH 8.5), 150 mM NaCl, 1% NP40, 5 mM EDTA supplemented with protease/phosphatase inhibitors. Nuclear fractions were obtained as detailed above. Lysates (500 μg of homogenate or 150 μg of nuclear fractions) were incubated overnight in rotation at 4 °C with 100 μl of protein-A sepharose beads (Sigma-Aldrich) crosslinked to LC3 antibody (MBL International, Woburn, MA, USA). For covalent crosslinking, beads were washed (2,500 r.p.m./5 min at 4 °C) with 0.2 M Borate/3 M NaCl buffer (pH 9.0) and crosslinked with 50 mM dimethyl pimelimidate in Borate buffer (30 min) in rotation at RT. Coupling reactions were stopped with 0.2 M ethanolamine (pH 8.0) for 2 h RT followed by incubation with 200 mM glycine (pH 2.5). Crosslinked beads were incubated with samples in rotation overnight at 4 °C. Bound proteins were eluted by boiling (95 °C for 5 min) in 2 × SDS–PAGE sample buffer. Immunoprecipitated (IP) proteins and original lysates (input) were resolved on a SDS–PAGE, and membranes were probed for bRAF, MEK, ERK, P-ERK and LC3. P-ERK1, P-ERK2, P-ERK1/2 or P-MEK1/2 bands obtained by immunoblotting were quantified using ImageJ and normalized to corresponding total ERK1, total ERK2, total ERK1/2 or total MEK1/2, respectively. Full scans of western blots are supplied in Supplementary Fig. S12. The antibody concentrations are supplied in Supplementary Table S1.

### Electron microscopy

Electron microscopy was performed in NIH/3T3 cells cultured in monolayers. Cells were collected and fixed in a buffer containing 2.5% glutaraldehyde and 2% paraformaldehyde in 100 mM sodium cacodylate (pH 7.43)^20^. Cells were post-fixed in 1% osmium tetroxide/sodium cacodylate buffer followed by fixation in 1% uranyl acetate. Cells were ethanol-dehydrated and embedded in LX112 resin (LADD Research Industries). Ultrathin sections were sequentially stained with uranyl acetate and lead citrate. Grids were visualized using a JEOL 1200CX II transmission electron microscope at 80 kV. Identification of autophagic vacuoles was done using established criteria^48^.

### Fluorescence microscopy

Cells on coverslips were fixed with a 4% paraformaldehyde solution, blocked and incubated with primary and corresponding secondary antibodies (Alexa Fluor 488 and/or Alexa Fluor 647 conjugated) (Invitrogen). Mounting medium contained DAPI (4',6-diamidino-2-phenylindole) to visualize the nucleus (Invitrogen). Images were acquired on a Leica DMI6000B microscope/ DFC360FX 1.4-megapixel monochrome digital camera (Leica Microsystems, Germany) using × 100 objective/1.4 numerical aperture. Images in each experiment were acquired at same exposure times within the same imaging session. Image slices/stacks of 0.2 μm thickness were captured and deconvolved using the Leica MetaMorph acquisition/analysis software. All images were prepared using Adobe Photoshop and subjected to identical post-acquisition brightness/contrast effects. Representative native and/or inverted images are shown. Quantification was performed in individual frames after deconvolution and thresholding using the ImageJ software (NIH) in a minimum of 20 cells per slide and a minimum of 50 cells from two or more experiments. Particle number was quantified with the ‘analyse particles’ function in threshold single sections with size (pixel^2^) settings from 0.1 to 10 and circularity from 0 to 1. Cellular fluorescence intensity was expressed as mean integrated density as a function of individual cell size. Nuclear fluorescence intensity was expressed as a function of nuclear size after the identification of the nuclear perimeter by the ImageJ ‘freehand selection tool’. Percentage colocalization was calculated using the JACoP plugin in single Z-stack sections of deconvolved images. Colocalization is shown in merged native images and/or as white pixels using the ‘colocalization finder’ plugin in ImageJ.

### RNA isolation and real-time PCR

Total RNA was isolated using the RNeasy Plus kit (Qiagen, USA) and genomic DNA eliminated via gDNA Eliminator spin columns. Total RNA (1 μg) was reverse transcribed into cDNA using Superscript II (Invitrogen), and real-time PCR (qPCR) was performed using Power SYBR Green PCR Master Mix (Applied Biosystems, UK) on a StepOnePlus Real-Time PCR System (Applied Biosystems, UK). Expression of *ERK1*, *ERK2*, *MEK1*, *MEK2*, *MKP3*, *PP2ACA, GATE16*, *Atg7*, *BECLIN1*, *LC3*, *LAMP-1 and LAMP-2A* were normalized to *TATA-binding protein* (*TBP*) and all reactions were in triplicate. The following primers were used: *ERK1* (forward (f)), 5′-TCCAAGGGCTACACCAAATC-3′, (reverse (r)) 5′-GTTTTCGAGGGCAGAGACTG-3′; *ERK2* (f) 5′-ACGGCATGGTTTGCTCTGCTTATG-3′, (r) 5′-TCATTTGCTCAATGGTTGGTGCCC-3′; *MEK1* (f) 5′-GTGAACTCACGTGGGGAGAT-3′, (r) 5′-CAGGAGGAGGAATGGGGTAT-3′; *MEK2* (f) 5′-CCCTACATCGTGGGCTTCTA-3′, (r) 5′-TGCATGATCTGGTGCTTCTC-3′; *MKP3* (f) 5′-TTGAATGTCACCCCCAATTT-3′, (r) 5′-CATCGTTCATGGACAGGTTG-3′; *PP2ACA* (f) 5′-TACTCCGAGGGAATCACGAG-3′, (r) 5′-TCGGATGTGATCCAGTGTGT-3′; *GATE-16* (f) 5′-TCTCGGGCTCTCAGATTGTT-3′, (r) 5′-GTGTTCTCTCCGCTGTAGGC-3′; *ATG7* (f) 5′-TCCGTTGAAGTCCTCTGCTT-3′, (r) 5′-CCACTGAGGTTCACCATCCT; *BECLIN1* (f) 5′-GGCCAATAAGATGGGTCTGA-3′, (r) 5′-GCTGCACACAGTCCAGAAAA-3′; *LC3* (f) 5′-ACAAAGAGTGGAAGATGTCCGGCT-3′, (r) 5′-TGCAAGCGCCGTCTGATTATCTTG-3′; *LAMP-1* (f) 5′-TAGTGCCCACATTCAGCATCTCCA-3′, (r) 5′-TTCCACAGACCCAAACCTGTCACT-3′; *LAMP-2A* (f) 5′-AGGTGCTTTCTGTGTCTAGAGCGT-3′, (r) 5′-AGAATAAGTACTCCTCCCAGAGCTGC-3′; *TBP* (f) 5′-GAAGCTGCGGTACAATTCCAG-3′, (r) 5′-CCCCTTGTACCCTTCACCAAT-3′; ZFP36 (f) 5′-TACGAGAGCCTCCAGTCGAT-3′, (r) 5′-GCGAAGTAGGTGAGGGTGAC-3′.

### Gene silencing with small interfering RNA

Cells were transiently transfected with 100 nM of small interfering RNA (siRNA) constructs or a non-related siRNA (siScramble) (Sigma-Aldrich) using Lipofectamine 2000 (Invitrogen) according to the manufacturer’s protocol. Cells were exposed to the silencing mix overnight following which silencing medium was replaced with fresh DMEM containing 10% NCS. The following siRNAs were used: siScramble (sense(s)) 5′-AAUUCUCCGAACGUGUCACGU-3′, (antisense (as)) 5′-ACGUGACACGUUCGGAGAAUU-3′; siATG7 (s) 5′-CUGUGAACUUCUCUGACGU-3′, (as) 5′-ACGUCAGAGAAGUUCACAG-3′; siATG5 (s) 5′-CCAUCAACCGGAAACUCAU-3′, (as) 5′-AUGAGUUUCCGGUUGAUGG-3′; siBeclin1 (s) 5′-CUGAGAAUGAAUGUCAGAA, (as) 5′-UUCUGACAUUCAUUCUCAG-3′; siATG4B (s) 5′-GAAAUCUUAUCCCUCUGAA-3′, (as) 5′-UUCAGAGGGAUAAGAUUUC-3′; siMKP3 (s) 5′-GUCUCUAGAUCCAGCAAUA-3′, (as) 5′-UAUUGCUGGAUCUAGAGAC-3′; siPP2ACA (s) 5′-CAGUAACUCCAAAGUGUCA-3′, (as) 5′-UGACACUUUGGAGUUACUG-3′. Silencing efficiency was determined by immunoblotting for the corresponding protein product or qPCR analyses for mRNA expression when the appropriate antibody was unavailable.

### Plasmids

Cyan fluorescence protein (CFP)-LC3, mutant CFP-LC3^ΔG^ in which the C-terminal glycine (120) is deleted^27^, WT ATG5 (Addgene plasmid #13095)^35^, mutant ATG5 K130R (Addgene plasmid #13096)^35^, ERK2 plasmids—WT-ERK2-HA (#8974)^32^, ERK2-L198A-HA (#8975)^32^, ERK2-L232A-HA (#8976)^32^, ERK2-L198A/L232A-HA (#8978)^32^, ERK2-Y261A-HA (#8977)^32^ and CD (ERK2-D319N-HA) (#8979)^32^ and pCGN-Elk1 (#27156)^49^ were acquired from Addgene (Cambridge, MA, USA) and transiently expressed by transfection with Lipofectamine 2000 (Invitrogen) as per the manufacturer’s instructions.

### Statistics

All numerical data arising from biochemical and cell fractionation analyses are represented as mean and s.e.m., and are from a minimum of three independent experiments unless otherwise stated. All imaging data are represented as mean and s.e.m, and represent data from at least 50 individual cells from two or more experiments. We determined the statistical significance of the difference between experimental groups in instances of single comparisons by the two-tailed unpaired Student’s *t*-test of the means. For multiple means comparisons, one-way analysis of variance (ANOVA) followed by Bonferroni *post hoc* test was used to determine statistical significance.

## Author contributions

N.M.-L. performed the experiments and analysed the data. D.A. assisted with immunofluorescence microscopy. S.S. and P.M. assisted with *in vivo* experiments. R.S. conceived the idea, designed the experiments, analysed and interpreted the data, and wrote the manuscript. All authors discussed and commented on the manuscript.

## Additional information

**How to cite this article:** Martinez-Lopez, N. *et al*. Autophagy proteins regulate ERK phosphorylation. *Nat. Commun.* 4:2799 doi: 10.1038/ncomms3799 (2013).

## Supplementary Material

Supplementary Information Supplementary Figures S1-S14 and Supplementary Table S1
