# Supplementary material for: Autophagy proteins regulate ERK phosphorylation
Source: Nat Commun. 2013 Nov 18;4:2799. doi: 10.1038/ncomms3799 (PMC3868163; doi:10.1038/ncomms3799)
Supplement: Supplementary Information — Supplementary Figures S1-S14 and Supplementary Table S1 [file ncomms3799-s1.pdf]

Supplementary Information

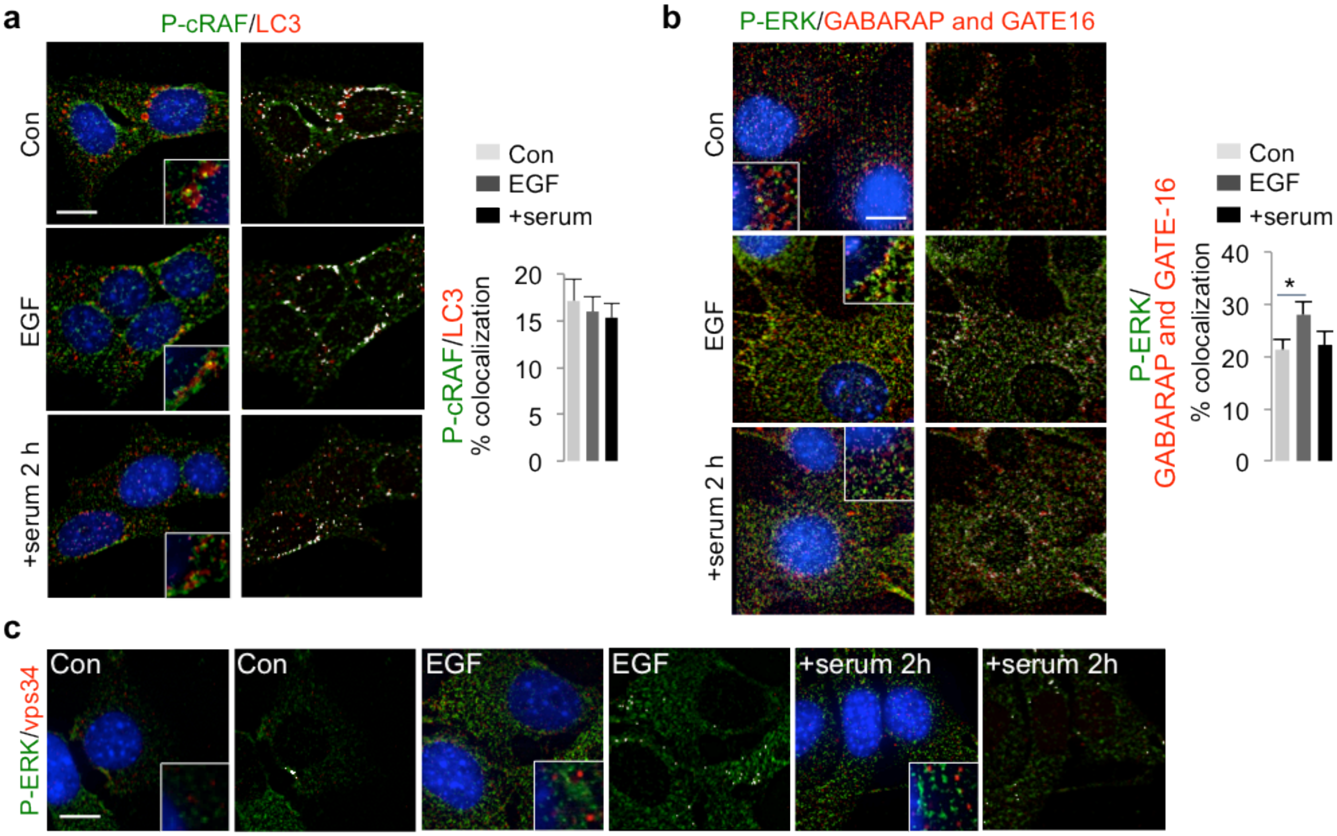

Supplementary Figure S1 (a) P-cRAF colocalizes with LC3 puncta. Immunofluorescence (IF) depicting colocalization of P-cRAF (green) and LC3 puncta (red) in NIH/3T3 cells treated with EGF for 10 min or serum for 2 h. Scale bar, 10 μm. The bars represent mean ± s.e.m. 60 cells analyzed from 2 experiments. Right panel depicts colocalization (white pixels) highlighted by “colocalization finder”. **(b)** P-ERK colocalizes with LC3 homologues GABARAP and GATE16. IF depicting colocalization of P-ERK (green) with GABARAP and GATE16 (red) in NIH/3T3 cells exposed to EGF for 10 min or serum for 2 h. Right panel depicts colocalization (white pixels) highlighted by “colocalization finder”. Scale bar, 10 μm. The bars represent mean ± s.e.m. *$P<0.05$ compared to Con; Student’s *t*-test, 60 cells analyzed from 2 experiments. **(c)** P-ERK colocalizes modestly with vps34. IF depicting colocalization of P-ERK (green) with vps34 (red) in NIH/3T3 cells exposed to EGF for 10 min or serum for 2 h. 60 cells analyzed from 2 experiments. Nuclei are in blue (DAPI). Scale bar, 10 μm.

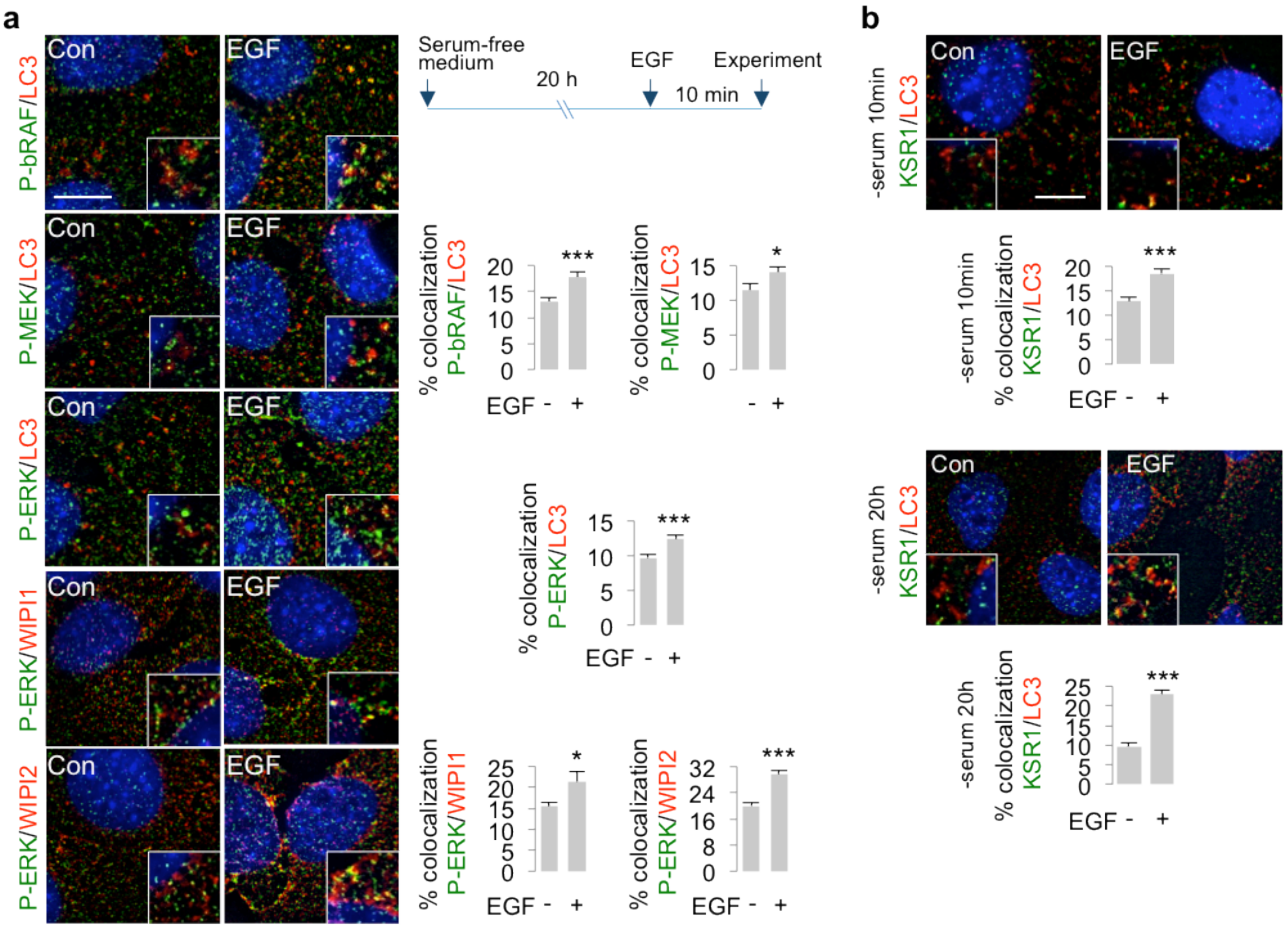

Supplementary Figure S2 (a) Components of the ERK signaling cascade colocalize with autophagy proteins in 20 h serum-deprivation cells. Immunofluorescence (IF) depicting colocalization of P-bRAF, P-MEK or P-ERK (green) with LC3 puncta, WIPI1 or WIPI2 (red) in 20 h serum-starved NIH/3T3 cells exposed to EGF for 10 min (scheme shown). Scale bar, 10 µm. The bars represent mean ± s.e.m. **P*<0.05, ****P*<0.001 compared to control (Con); Student's *t*-test, 50 cells analyzed from 2 experiments. **(b)** ERK scaffold complex protein KSR1 (kinase suppressor of Ras) colocalizes with LC3 puncta. IF depicting colocalization of KSR1 with LC3 in 10 min **(b, top)** or 20 h **(b, bottom)** serum-starved NIH/3T3 cells treated or not with EGF for 10 min. Scale bar, 10 µm. The bars represent mean ± s.e.m. ****P*<0.001 compared to Con; Student's *t*-test, 50 cells analyzed from 2 experiments. Nuclei are in blue (DAPI).

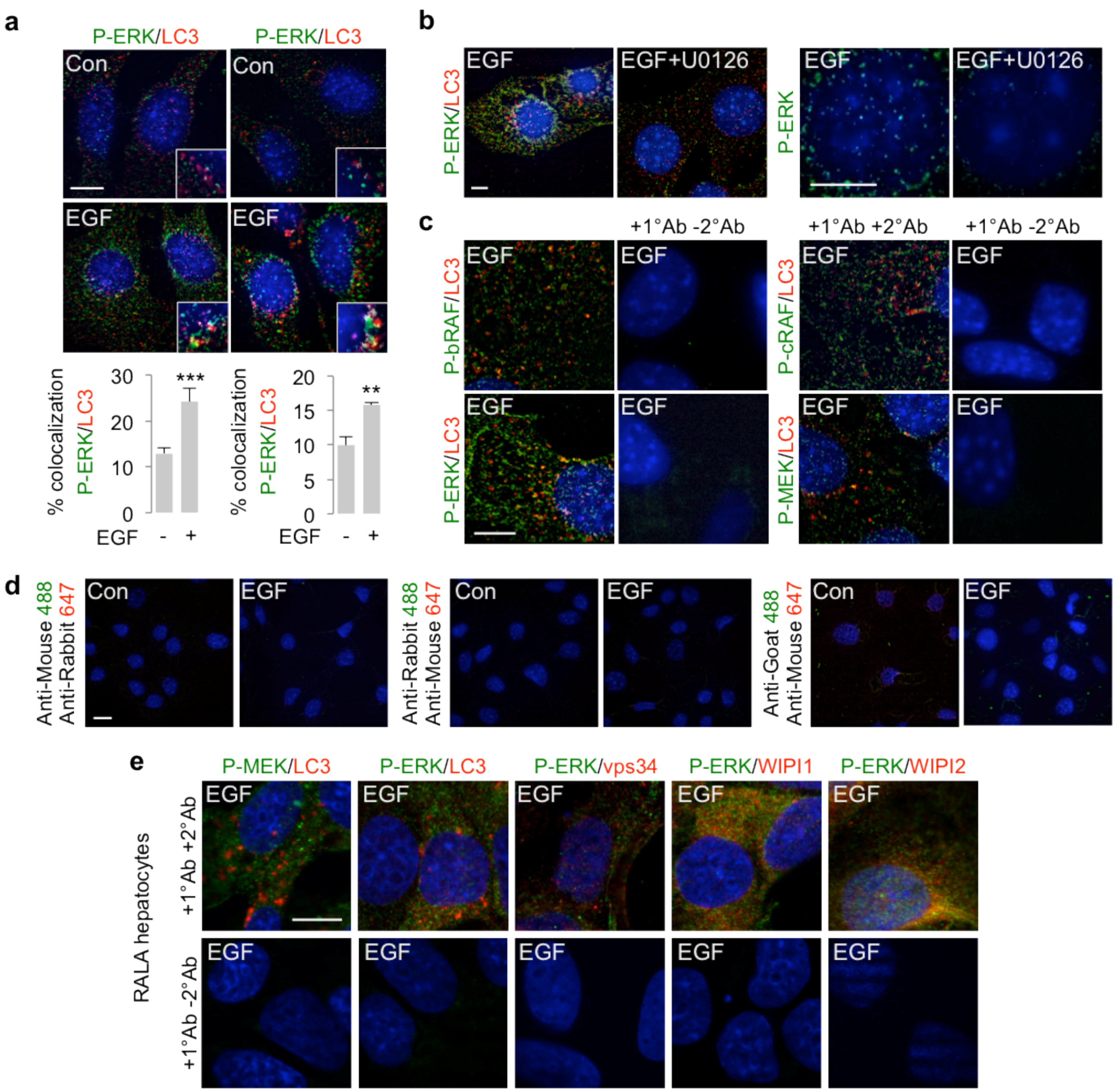

Supplementary Figure S3 Primary antibody (1°Ab) and fluorescent-tagged secondary antibody specificities. **(a)** Validation of P-ERK/LC3 colocalizations. Immunofluorescence (IF) depicting colocalization of **(a, left)** P-ERK (rabbit 1°Ab) (green) and LC3 (mouse 1°Ab) (red), and **(a, right)** P-ERK (mouse 1°Ab) (green) and LC3 (rabbit 1°Ab) (red) in NIH/3T3 cells treated or not with EGF (10 min). Scale bar, 10 μm. Bars represent mean ± s.e.m. ***P*<0.01, ****P*<0.001 compared to Con; Student's *t*-test, 60 cells analyzed from 2 experiments. **(b)** Validation of P-ERK fluorescence. IF depicting reduced **(b, left)** cytoplasmic or **(b, right)** nuclear P-ERK (green) signal in 2 h U0126-pretreated NIH/3T3 cells exposed to EGF (10 min). LC3 is in red. 60 cells analyzed from 2 experiments. Scale bar, 5 μm. **(c)** Antibody specificity in NIH/3T3 cells. IF in EGF-treated cells exposed to the indicated primary antibodies and corresponding secondary antibodies (+1°Ab +2°Ab) or only primary antibodies (+1°Ab -2°Ab). Scale bar, 10 μm. **(d)** IF in EGF-treated NIH/3T3 cells exposed to secondary antibodies alone. Scale bar, 10 μm. **(e)** Antibody specificities in RALA hepatocytes. IF in EGF-treated RALA cells exposed to the indicated primary antibodies and corresponding secondary antibodies (+1°Ab +2°Ab) or only primary antibodies (+1°Ab -2°Ab). Scale bar, 10 μm. Nuclei are in blue (DAPI).

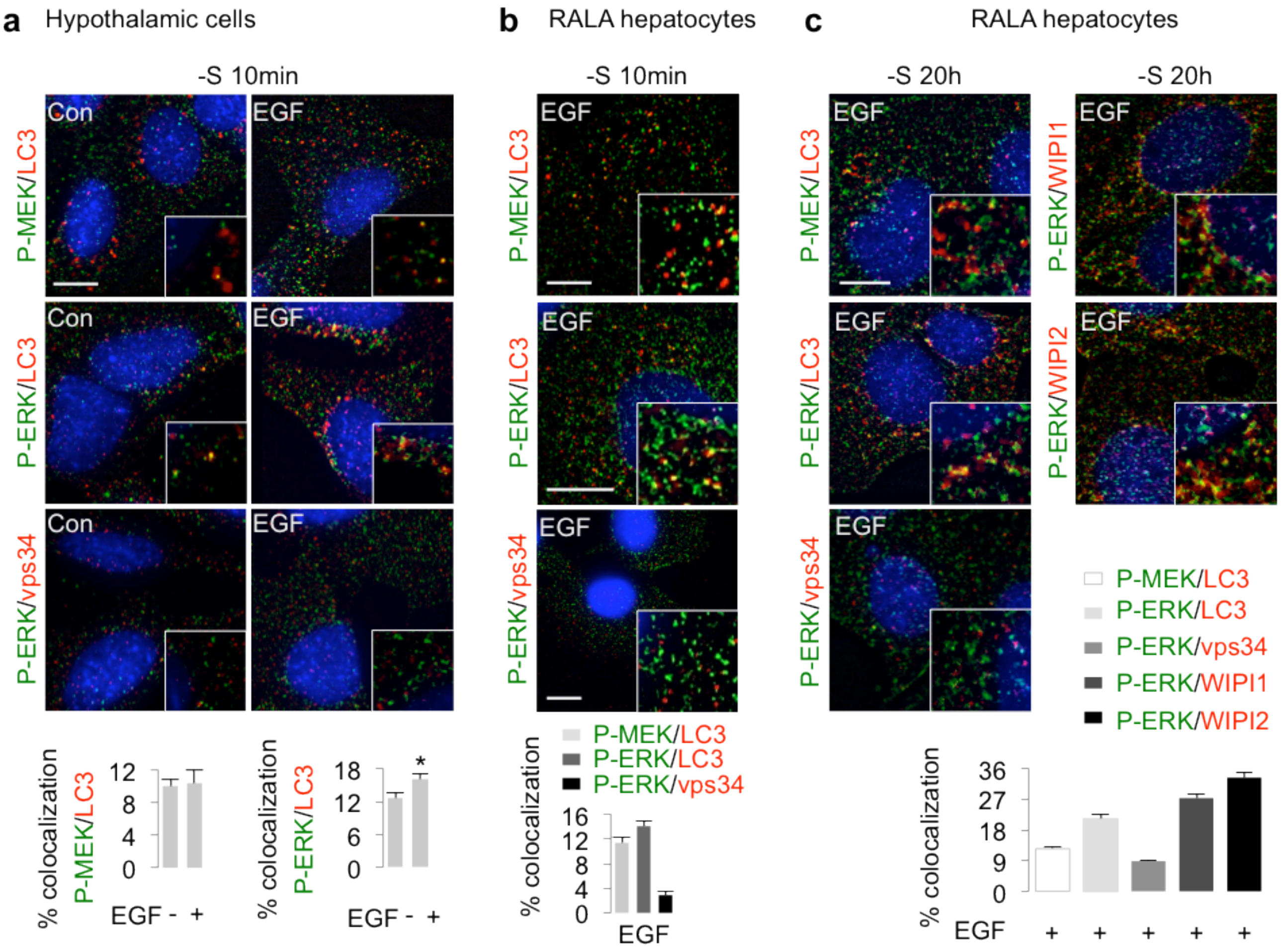

Supplementary Figure S4 Immunofluorescence (IF) depicting colocalization of P-MEK (green) and P-ERK (green) with LC3 puncta (red) or vps34 (red) in **(a)** hypothalamic cells and **(b)** 10 min serum-starved RALA rat hepatocytes treated with EGF (10 min). Scale bars, 10 μm. For panels, **a** and **b**, bars represent mean ± s.e.m. **P*<0.05 compared to EGF-untreated cells; Student's *t*-test, 60 cells were analyzed from 2 experiments. **(c)** IF showing colocalization of P-MEK (green) and P-ERK (green) with LC3-II, vps34, WIPI1 or WIPI2 (red) in 20 h serum-starved RALA hepatocytes treated with EGF (10 min). Scale bar, 10 μm. The bars represent mean ± s.e.m. 50 cells were analyzed from 2 experiments. Nuclei are in blue (DAPI).

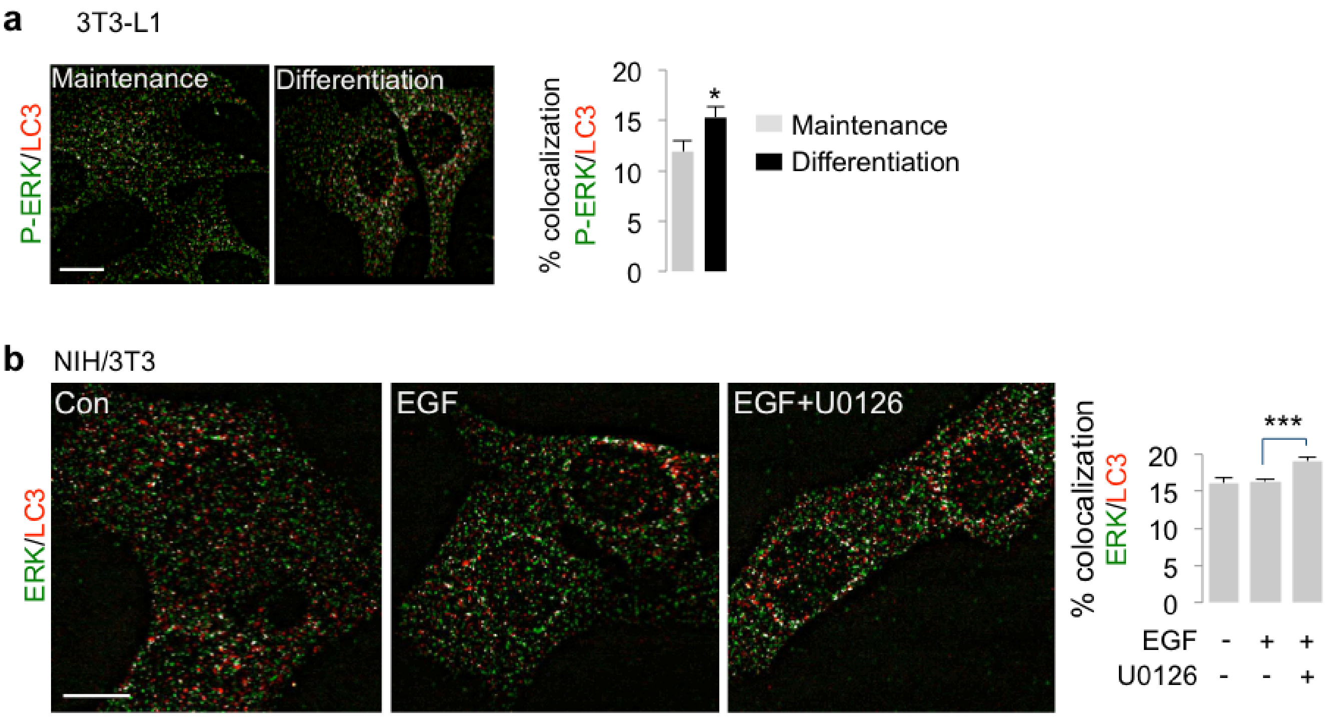

Supplementary Figure S5 (a) IF showing colocalizations of P-ERK (green) and LC3 puncta (red) in 3T3-L1 preadipocytes cultured in regular maintenance medium or in adipocyte differentiation medium (2 h). Bottom panel – colocalization (white pixels) highlighted by “colocalization finder”. Scale bar, 10 µm. The bars represent mean ± s.e.m. **P*<0.05 compared to EGF-untreated cells; Student’s *t*-test, 60 cells analyzed from 2 experiments. **(b)** ERK phosphorylation is not a requirement for ERK to colocalize with LC3-II. IF showing colocalization of ERK (green)/LC3 (red) in 2 h U0126-pretreated NIH/3T3 cells exposed to EGF (10 min). Colocalization (white pixels) highlighted by “colocalization finder”. Scale bar, 10 µm. The bars represent mean ± s.e.m. ****P*<0.001 compared to EGF-untreated cells; Student’s *t*-test, 60 cells analyzed from 2 experiments.

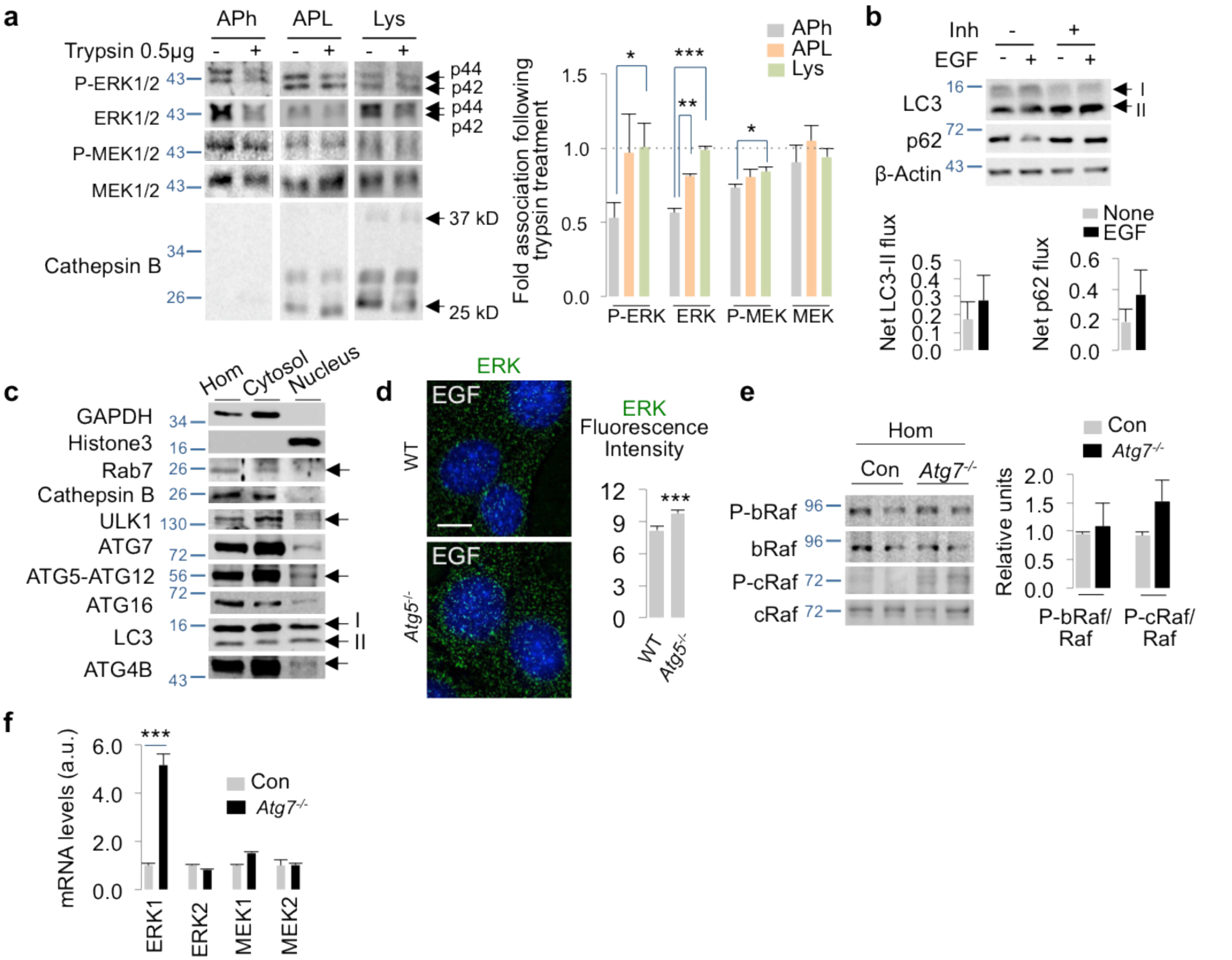

Supplementary Figure S6 (a) MEK and ERK localize to the extra-luminal face of autophagosomes (APh). Immunoblots show P- and total MEK and ERK in APh, autophagolysosome (APL) and lysosome (Lys) fractions isolated from mice livers and untreated (-) or treated with trypsin (0.5 µg for 15 min). Bars represent mean ± s.e.m. **P*<0.05, ***P*<0.01, ****P*<0.001 compared to trypsin-induced dissociation in APh; Student's *t*-test, n = 3. The p44 and p42 forms of ERK are indicated. **(b)** Exposure to EGF does not affect net LC3-II or p62 flux. Immunoblots for LC3, p62 and β-actin in lysosomal inhibitor (Inh)-pretreated NIH/3T3 cells (2 hours) exposed to EGF (10 min). Bars represent mean ± s.e.m. Student's *t*-test, n = 5. **(c)** Presence of autophagy proteins in the nucleus. Immunoblots for ULK1, ATG7, ATG5-ATG12, ATG16, LC3 and ATG4B, and soluble nuclear marker histone3, soluble cytoplasmic marker GAPDH, endosomal marker Rab7 and lysosomal marker Cathepsin B in homogenate (Hom), cytosol and nuclear fractions from fed livers, n = 5. Rab7, ULK1, ATG5-ATG12, LC3-I and membrane-associated LC3-II are indicated. **(d)** Deleting *Atg5* increases nuclear total ERK content. IF for total ERK in EGF-treated WT and $Atg5^{-/-}$ MEFs. Scale bar, 10 µm. ****P*<0.001 compared to WT cells; Student's *t*-test, 50 cells analyzed from 2 experiments. **(e)** Loss of ATG7 does not affect phosphorylation of bRAF or cRAF. Immunoblots for P- and total bRAF and cRAF from Hom of Con and $ATG7^{-/-}$ livers. Bars represent mean ± s.e.m. Student's *t*-test, n = 4. **(f)** Loss of ATG7 increases ERK1 expression. Messenger RNA levels for *ERK1*, *ERK2*, *MEK1* and *MEK2* genes in Con and $Atg7^{-/-}$ livers. Bars represent mean ± s.e.m. ****P*<0.001 compared to Con; Student's *t*-test, n = 4.

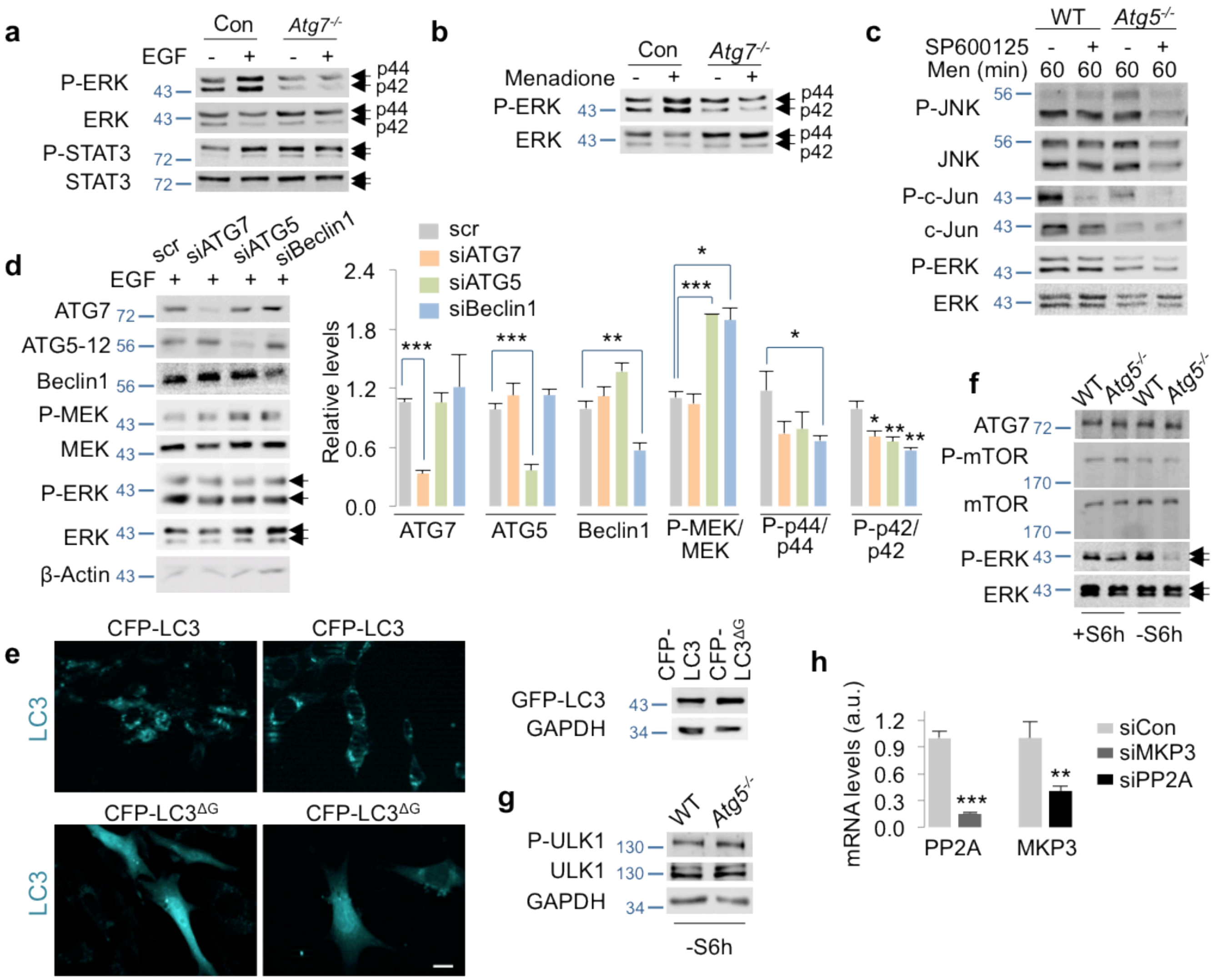

Supplementary Figure S7 **(a)** *Atg7*$^{-/-}$ livers exhibit decreased ERK phosphorylation, but maintained STAT3 phosphorylation, in response to EGF. Immunoblots for P- and total ERK or STAT3 levels in fresh liver explants exposed to EGF, n = 4. **(b)** *Atg7*$^{-/-}$ livers display decreased ERK phosphorylation in response to menadione. Immunoblots for P- and total ERK levels in liver explants exposed to menadione, n = 4. **(c)** Blocking JNK/c-jun signaling does not modify ERK phosphorylation in WT or *Atg5*$^{-/-}$ MEFs. Immunoblots for P- and total JNK, c-jun and ERK in WT and *Atg5*$^{-/-}$ MEFs pretreated with JNK inhibitor SP600150 (30 min) and exposed to menadione (Men), n = 3. **(d)** Transiently silencing *Atg* genes decreases EGF-induced ERK phosphorylation. Immunoblots for ATG7, ATG5, Beclin1, P- and total MEK and ERK, and β-actin in total lysates from NIH/3T3 cells transiently transfected with scr, siATG7, siATG5 or siBeclin1, and treated with EGF (10 min). The p44/p42 forms of ERK are indicated. Bars represent mean ± s.e.m. **P*<0.05, ***P*<0.01, ****P*<0.001 compared to scr-transfected cells; Student's *t*-test, n = 4. **(e)** Immunofluorescence **(e, left)** and immunoblots **(e, right)** displaying equivalent expression of CFP in NIH/3T3 cells transfected with CFP-LC3- and CFP-LC3^{ΔG}, n = 8. Scale bar, 10 μm. **(f)** Serum-starved *Atg5*$^{-/-}$ MEFs have decreased P-ERK levels but unaffected P-mTOR levels. Immunoblots for P- and total ERK and mTOR in 6 h serum-fed or serum-starved WT and *Atg5*$^{-/-}$ MEFs, n=3 **(g)** Serum-starved WT and *Atg5*$^{-/-}$ MEFs exhibit equivalent ULK1 phosphorylation. Immunoblots for P- and total ULK1 in 6 h serum-starved WT and *Atg5*$^{-/-}$ MEFs, n=3. **(h)** mRNA levels depicting MKP3 and PP2A knockdowns in *Atg5*$^{-/-}$ MEFs. Bars represent mean ± s.e.m. ***P*<0.01, ****P*<0.001 compared to controls; Student's *t*-test, n = 5.

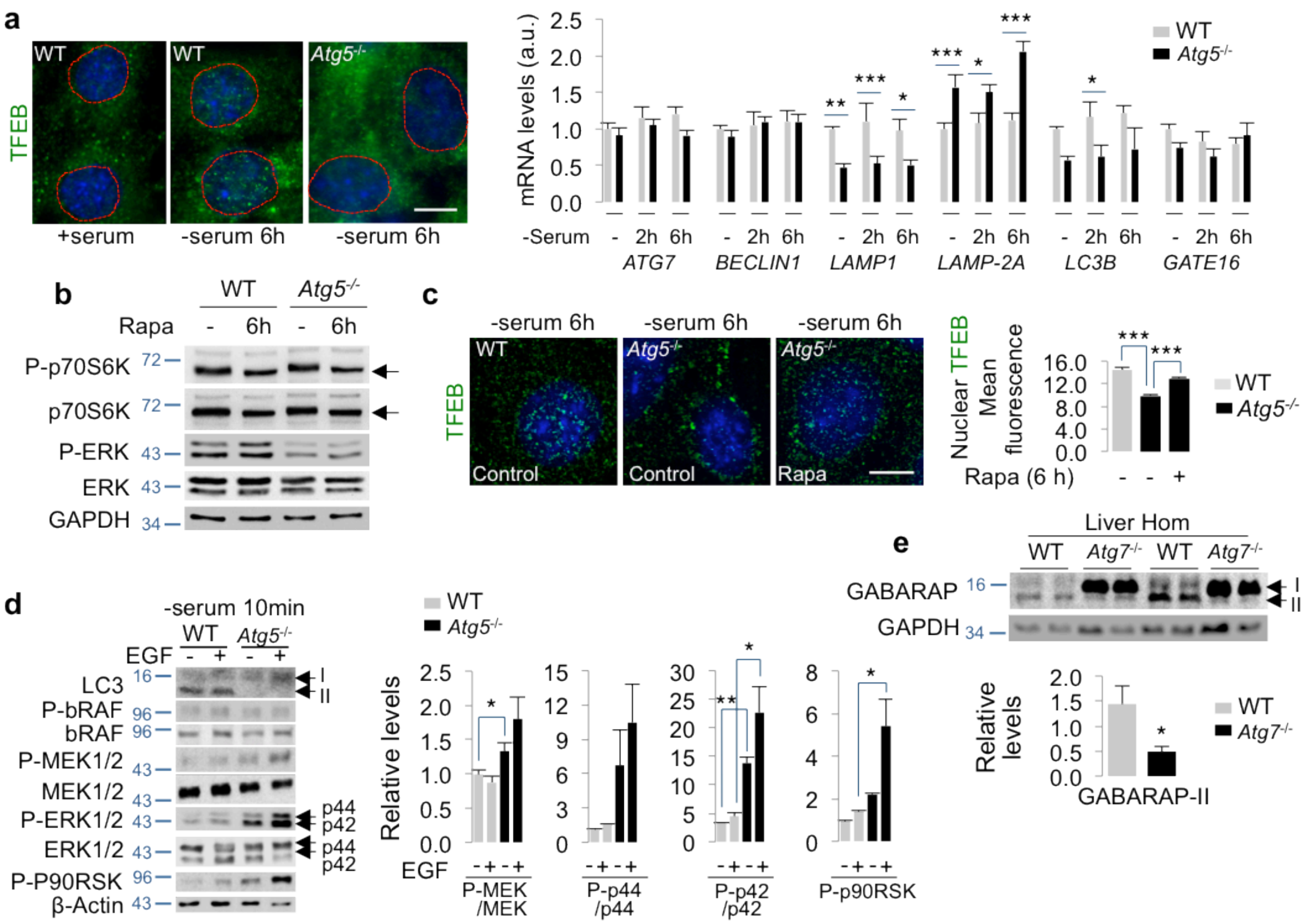

Supplementary Figure S8 (a) Decreased ERK phosphorylation in *Atg5*$^{-/-}$ MEFs associates with reduced nuclear TFEB levels and decreased *LC3B* and *LAMP1* gene expression. Immunofluorescence (IF) for TFEB in 6 h serum-fed or serum-starved WT and *Atg5*$^{-/-}$ MEFs, n=3. mRNA levels for indicated genes in serum-fed, 2 h and 6 h serum-starved WT and *Atg5*$^{-/-}$ MEFs. Scale bar, 10 µm. Bars represent mean ± s.e.m. **P*<0.05, ***P*<0.01, ****P*<0.001; ANOVA-Bonferroni *post hoc* test, n = 3. **(b)** Immunoblots for P- and total p70S6K and ERK, and GAPDH in WT and *Atg5*$^{-/-}$ MEFs treated or not with rapamycin (Rapa), n=3. **(c)** IF for TFEB (stained in green) in 6 h serum starved WT and *Atg5*$^{-/-}$ MEFs exposed to Rapa or not. Scale bar, 10 µm. Bars represent mean ± s.e.m. ****P*<0.001; Student's *t*-test, 50 cells analyzed from 2 experiments. **(d)** Increased MEK and ERK phosphorylation in *Atg5*$^{-/-}$ MEFs during early nutrient deprivation (-serum 10 min). Immunoblots for LC3, P- and total bRAF, P- and total MEK and ERK, P-p90RSK and β-actin from 10 min serum-deprived WT and *Atg5*$^{-/-}$ MEFs in presence or absence of EGF. LC3-I and LC3-II are indicated. Bars represent mean ± s.e.m. **P*<0.05, ***P*<0.01; Student's *t*-test, n = 3. **(e)** GABARAP-II levels are reduced in *Atg7*$^{-/-}$ livers. Immunoblots showing GABARAP and GAPDH levels in homogenates from WT and *Atg7*$^{-/-}$ livers. Bars represent mean ± s.e.m. *P*<0.05; Student's *t*-test, n = 4. GABARAP-I and GABARAP-II are indicated. Nuclei are in blue (DAPI).

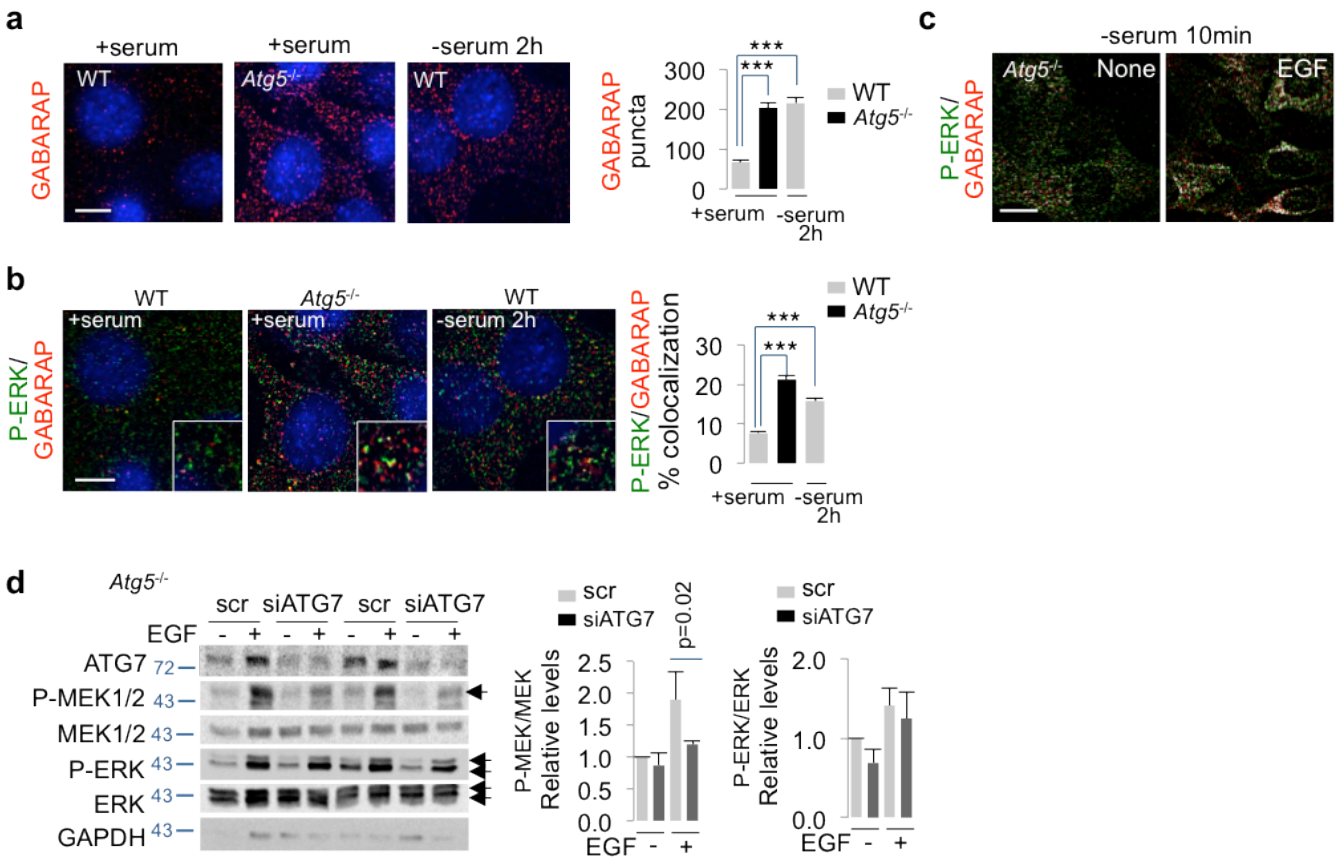

Supplementary Figure S9 (a) Increased GABARAP puncta in serum-fed *Atg5*$^{-/-}$ MEFs. Immunofluorescence (IF) depicting GABARAP puncta (red) in serum-fed WT and *Atg5*$^{-/-}$ MEFs, and in 2 h serum-starved WT MEFs. Scale bar, 10 µm. Bars represent mean ± s.e.m. ****P*<0.001; Student's *t*-test, 60 cells analyzed from 2 experiments. **(b)** Increased P-ERK/GABARAP colocalization in serum-fed *Atg5*$^{-/-}$ MEFs. IF showing P-ERK (green) and GABARAP (red) in WT and *Atg5*$^{-/-}$ MEFs in presence or absence of serum (2 h). Scale bar, 10 µm. Bars represent mean ± s.e.m. ****P*<0.001; Student's *t*-test, 60 cells analyzed from 2 experiments. **(c)** Increased P-ERK/GABARAP colocalization in EGF-treated *Atg5*$^{-/-}$ MEFs. IF for P-ERK (green) and GABARAP (red) in serum-starved (10 min) *Atg5*$^{-/-}$ MEFs treated with or without EGF (10 min). Colocalization (white pixels) highlighted by "colocalization finder" application. 60 cells were analyzed from 2 experiments. Scale bar, 10 µm. **(d)** Depleting ATG7 in *Atg5*$^{-/-}$ MEFs compromises EGF-induced MEK phosphorylation. Immunoblots for ATG7, P- and total MEK and ERK, and GAPDH from *Atg5*$^{-/-}$ MEFs transfected with scr or siATG7 in presence or absence of EGF. Bars represent mean ± s.e.m. **P*<0.05; Student's *t*-test, n = 3.

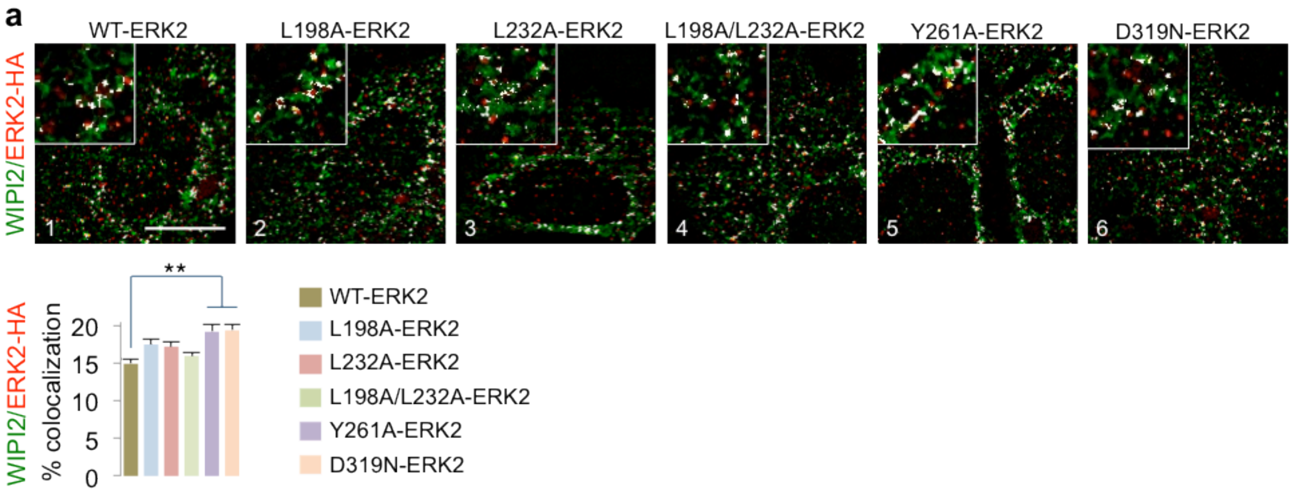

b

>gi|16716341|ref|NP_444299.1| **autophagy protein 5 [Mus musculus]**
MTDDKDVLRDVWFGRIPTCFTLYQDEITEREAEPYYLLLPRVSYLTLVTDKVKKHFQKVMRQEDVSEIWFEYEGTPLKWHYPIGLLFDL
LASSSALPWNITVHFKSFPEKDLLHCPSKDAVEAHFMSCMKEADALKHKSQVINEMQKKDHKQLWMGLQNDRFDQFWAINRKLMEY
PPEENGFRYIPFRIYQTTTERPFIQKLFRPVAADGQLHTLGDLLREVCPSAVAPEDGEKRSQVMIHGIEPMLETPLQWLSEHLSYPDNF
LHISIVPQPTD

>gi|145966752|ref|NP_080493.2| **ubiquitin-like protein ATG12 [Mus musculus]**MSEDSEVVLQLPSAPVGAGGESLPELSPETATPEPPSSAAVSPGTEEPPGDTKKKIDILLKAVGDTPIMKTKKWAVERTRTI
QGLIDFI**KK**F**LKL**VASEQLFIYVNQSFAPSPDQEVGTLYECFGSDGKLVLHYCKSQAWG

>gi|13385664|ref|NP_080436.1| **microtubule-associated proteins 1A/1B light chain 3B [Mus musculus]**
MPSEKTFKQRRSFEQRVEDVRLIREQHPTKIPVIIERYKGEKQLPVLDKTKFLVPDHVNMSELI**K**II**RRRLQL**NANQAFFLLVNGHSM
VSVSTPISEVYESERDEDGFLYMVYASQETFGTAMAV

>gi|9789961|ref|NP_062723.1| **gamma-aminobutyric acid receptor-associated protein [Mus musculus]**
MKFVYKEEHPFEKRRSEGEKIRKKYPDRVPVIVEKAPKARIGDLDKKKYLVPSDLTVGQFYFLIRKRIHLRAEDALFFFVNNVIPPTSAT
MGQLYQEHHEEDFFLYIAYSDESVYGL

>gi|22122367|ref|NP_666052.1| **WD repeat domain phosphoinositide-interacting protein 1 [Mus musculus]**
MEAEAADAPPGRVEAALSCFSFNQDCTSLAIGTKAGYKLFSLSSVEQLDQVHGSNEIPDVYIVERLFSSSLVVVVSHTKPRQMNVYHF
KKGTEICNYSYSSNILSIRLNRQRLLVCLEESIYIHNIKDMKLLKTVLDIPSNPTGLCALSINHSNSYLAYPGSQSTGEIVLYDGNSLKTVC
TIAAHEGTLAAITFNSSGSKLASASEKGTVIRVFSVPEGQKLYEFRRGMKRYVTISSLVFSMDSQFLCASSNTETVHIFKMEHLTDSRPE
EPSTWSGYMGKMFMAATNYLPAQVSDMMNQDRAFATGRLNFSGQKNICTLSTIQKLPRLLVASSDGHLYIYNLDPQDGGECVLIKTH
SLLSSGTTEENKENDLRPSLPPSYAATVARPSTSAASTVPGYSEDGGALRGEVIPEHEFATGPVCLDDENEFPPIILCRGSQKGKTKQS

>gi|30409972|ref|NP_848485.1| **WD repeat domain phosphoinositide-interacting protein 2 [Mus musculus]**
MNLASQSGEAGAGQLLFANFNQDNTSLAVGSKSGYKFFSLSSVDKLEQIYECTDTEDVCIVERLFSSSLVAIVSLKAPRKLKVCHFKKG
TEICNYSYSNTILAVKLNRQRLIVCLEESLYIHNIRDMKVLHTIRETPPNPAGLCALSINNDNCYLAYPGSASIGEVQVFDTINLRAANMIP
AHDSPLAALAFDASGTKLATASEKGTVIRVFSIPEGQKLFEFRRGVKRCVSICSLAFSMDGMFLSASSNTETVHIFKLEAVREKPPEEPT
TWTGYFGKVLMASTSYLPSQVTEMFNQGRAFATVRLPFCGHKNICSLTTIQKIPRLLVGASDGYLYMYNLDPQEGGECALMRQHRLD
GSMETTSEIVDSASHDCPLATQTYGTAAAKGAYVPSSPTRLGKGQDANLEAYTDDLGAVGGACLEDEASALRLDEDSEHPPMILRTD

Supplementary Figure S10 Mutations in ERK2 kinase-docking domains do not affect ERK2/WIPI2 colocalization. **(a)** Immunofluorescence showing colocalization of WT-ERK2-HA (red), FRS ERK2 mutants (L198A-, L232A-, L198A/L232A-, Y261A-ERK2-HA) (red) or common docking (CD) mutant (D319N-ERK2-HA) (red) with WIPI2 (green) in EGF-treated NIH/3T3 cells. Scale bar, 10 μm. Bars represent mean ± s.e.m. $^{**}P<0.01$ compared to WT-ERK2-transfected cells; Student's *t*-test, 50 cells analyzed from 2 experiments. **(b)** ATG12 and LC3B display substrate D-domains required to interact with ERK2. Amino acid sequences for ATG5, ATG12, LC3B, GABARAP, WIPI1 and WIPI2 indicating the presence of D-domains, i.e., basic residues (K, lysine or R, arginine) followed by a hydrophobic LXL motif[47] in ATG12 and LC3B. D-domains in ATG12 and LC3B are in larger fonts and underlined in green.

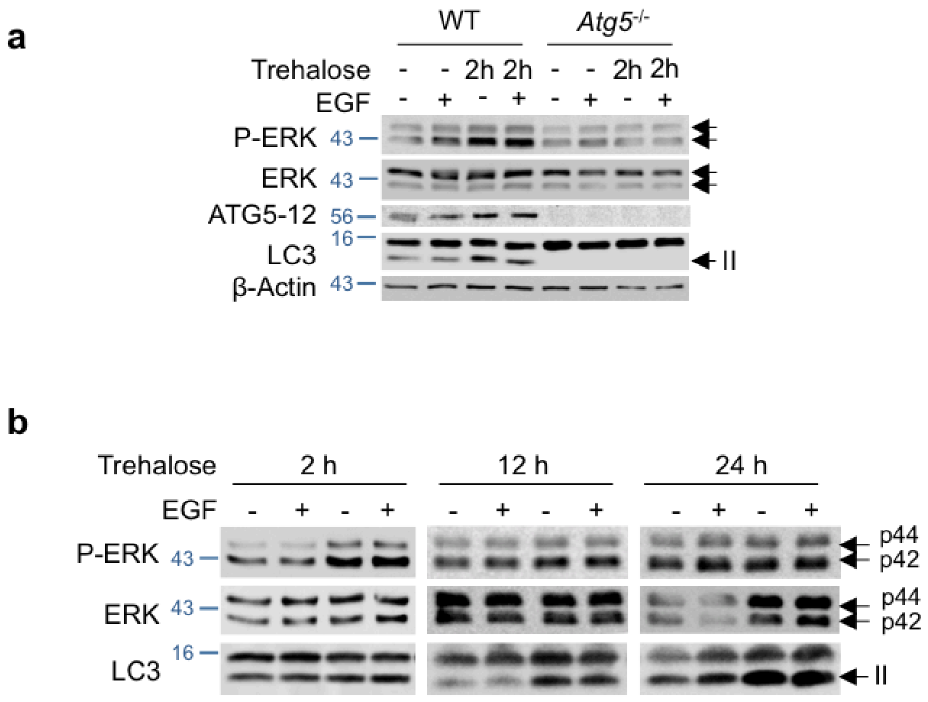

Supplementary Figure S11 (a) Acute trehalose exposure increases ERK phosphorylation that requires ATG5. Immunoblots for P- and total ERK, ATG5-ATG12, LC3 and β-actin in WT and *Atg5*$^{-/-}$ MEFs exposed to EGF in presence or absence of trehalose for 2 h, n = 3. **(b)** Prolonged exposure of cells to trehalose fails to increase ERK phosphorylation. Immunoblots for P- and total ERK, and LC3 in WT and *Atg5*$^{-/-}$ MEFs exposed to EGF in presence or absence of trehalose for 12 h and 24 h, n = 3.

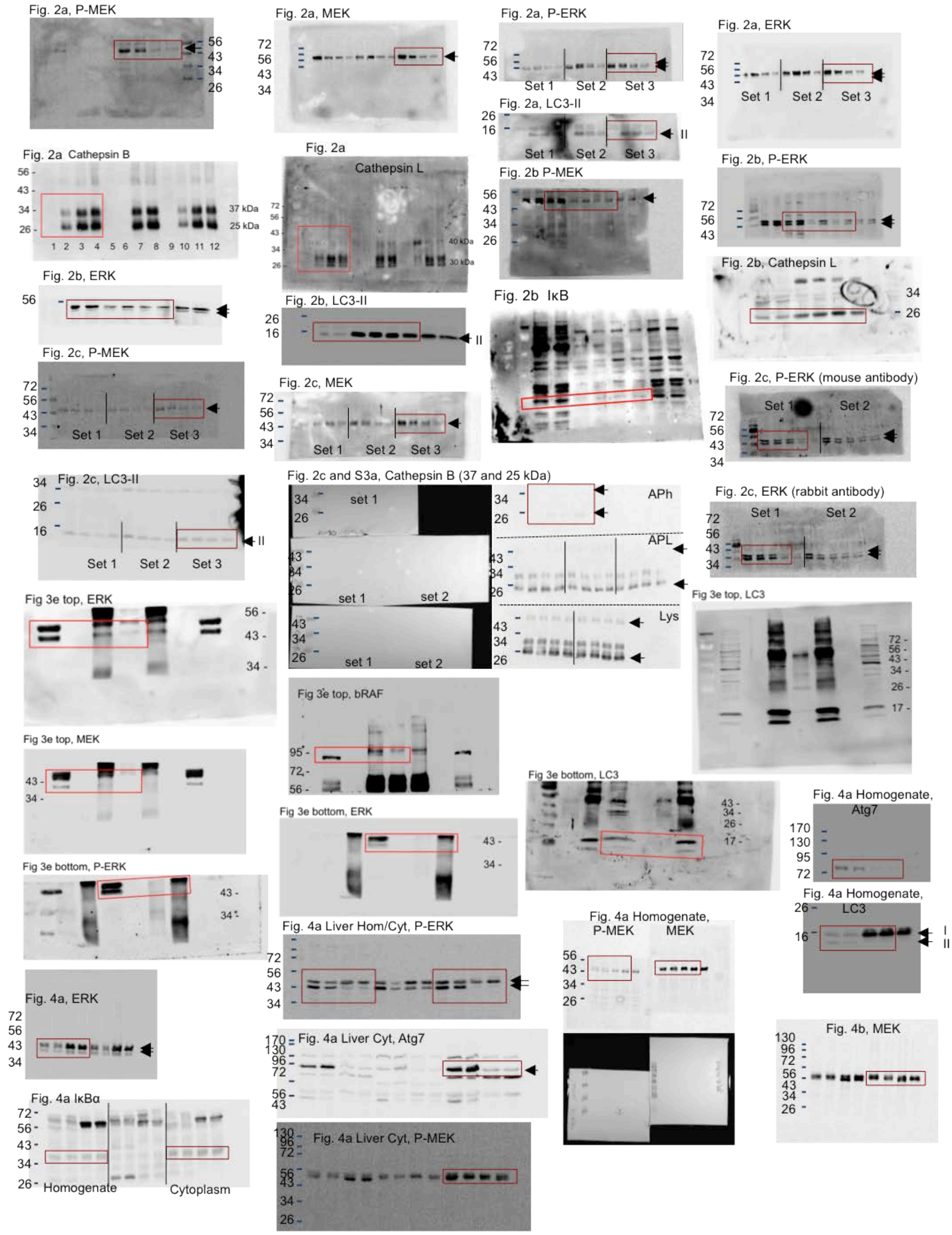

Supplementary Figure S12. Full-length uncropped images of gels displayed in the Main figures

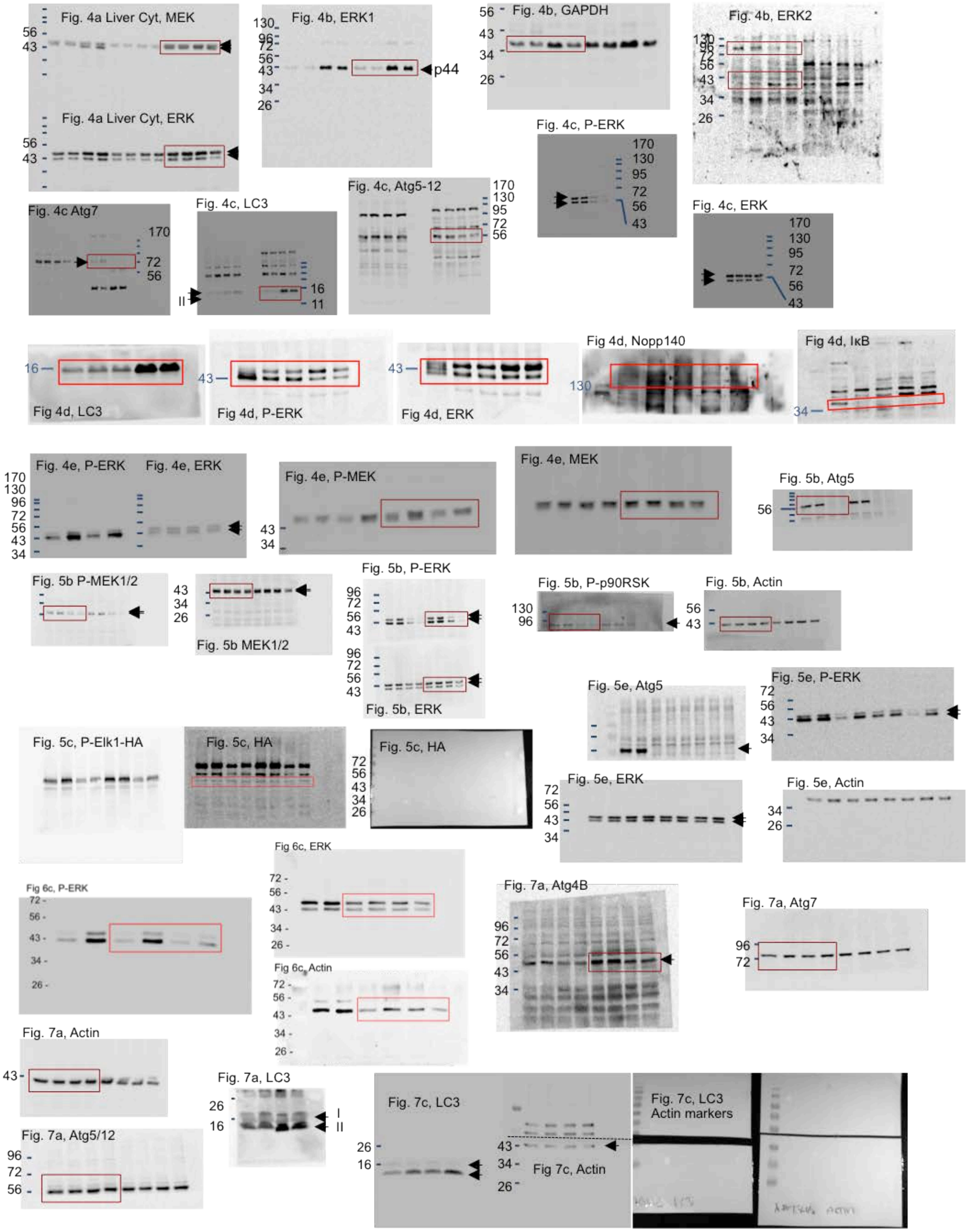

Supplementary Figure S13. Full-length uncropped images of gels displayed in the Main figures

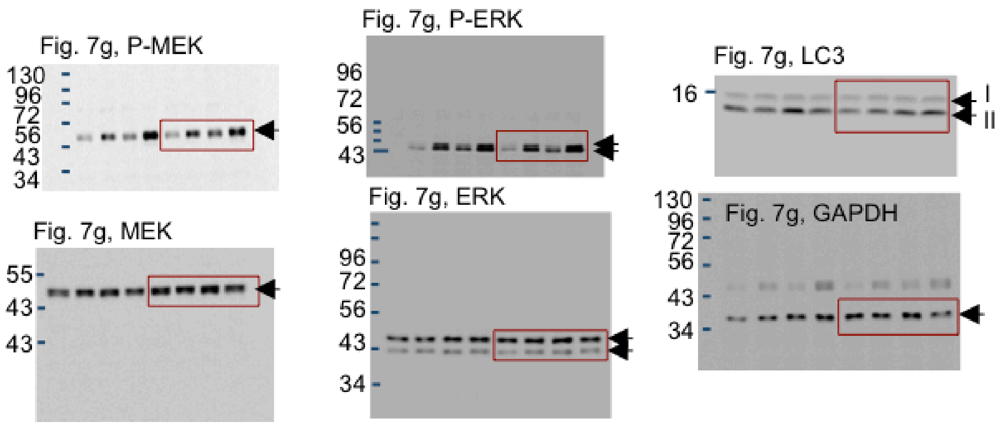

Supplementary Figure S14. Full-length uncropped images of gels displayed in the Main figures

Supplementary Table S1. List of antibodies used in this study

			Western blot	IF	IP
**Primary Antibody**	**Source**	**Catalog #**	**Dilution**	**Dilution**	
**β-Actin**	Abcam	ab8227	1:1000		
**ATG16**	Santa Cruz	sc-70133	1:1000	1:200	
**ATG4B**	Cell Signaling	5299	1:1000		
**ATG5-ATG12**	Novus Biologicals	NB110-53818	1:1000	1:200	
**ATG7**	Cell Signaling	2631	1:1000	1:50	
**Beclin1**	BD Biosciences	612112	1:500	1:200	
**P-bRAF**	Cell Signaling	2692	1:1000	1:250	
**bRAF**	Cell Signaling	9434	1:1000		
**Cathepsin B**	Santa Cruz	sc-6493	1:500		
**Cathepsin L**	Santa Cruz	sc-6498	1:500		
**P-c-Jun**	Santa Cruz	sc-822	1:500		
**c-Jun**	Santa Cruz	sc-45	1:500		
**P-cRAF**	Cell Signaling	9427	1:1000	1:250	
**cRAF**	Cell Signaling	9422	1:1000		
**P-ELK-1**	Cell Signaling	9181	1:1000		
**P-ERK**	Cell Signaling	9101	1:1000	1:500	
**ERK**	Cell Signaling	9102	1:1000	1:500	
**P-ERK**	Cell Signaling	9106	1:1000	1:500	
**ERK**	Cell Signaling	4696	1:1000	1:500	
**ERK1**	Cell Signaling	4372	1:1000		
**ERK2**	Cell Signaling	9108	1:1000		
**GABARAP/GATE-16**	Santa Cruz	sc-28938	1:500	1:50	
**GAPDH**	Abcam	ab8245	1:1000		
**HA**	Cell Signaling	2367	1:1000		
**IKB**	Santa Cruz	sc-371	1:1000		
**JL-8**	Clontech	632380	1:10,000		
**P-JNK**	Santa Cruz	sc-62254	1:1000		
**JNK**	Santa Cruz	sc-571	1:1000		
**KSR1**	Cell Signaling	4640	1:1000	1:250	
**LC3**	Cell Signaling	2775	1:1000	1:50	
**LC3**	MBL	PM036	1:1000		1:200
**LC3**	MBL	M152-3		1:200	
**P-MEK**	Cell Signaling	9154	1:1000	1:250	
**MEK**	Cell Signaling	9122	1:1000	1:250	
**P-mTOR**	Cell Signaling	2971	1:1000		
**mTOR**	Cell Signaling	2972	1:1000		
**Nopp140**	Dr. U. Thomas Meier, AECOM	-	1:500		
**P-p90RSK**	Cell Signaling	9344	1:1000		
**P-p70S6K**	Cell Signaling	9205	1:1000		
**p70S6K**	Cell Signaling	9202	1:1000		
**Rab7**	Santa Cruz	sc-10767	1:1000		
**P-STAT3**	Cell Signaling	9145	1:1000		
**STAT3**	Cell Signaling	4904	1:1000		
**TFEB**	Santa Cruz	sc-48784		1:100	
**P-ULK1**	Cell Signaling	5869	1:1000	1:100	
**ULK1**	Novus Biologicals	NB110-74844	1:500		
**Vps34**	Invitrogen	382100		1:200	
**WIPI-1**	Abcam	ab128901		1:100	
**WIPI-2**	Abcam	ab105459		1:100	
**STAT3**	Cell Signaling	4904	1:1000		
**TFEB**	Santa Cruz	sc-48784		1:100	
**P-ULK1**	Cell Signaling	5869	1:1000	1:100	
**Secondary Antibody**	**Source**	**Catalog #**	**Dilution**	**Dilution**	
**HRP-Rabbit anti-Mouse**	Invitrogen	61-6520	1:5000		
**HRP-Goat anti-Rabbit**	Invitrogen	65-6120	1:5000		
**HRP-Rabbit anti-Goat**	Invitrogen	61-1620	1:5000		
**Goat anti-Rabbit 448**	Invitrogen	A11008		1:500	
**Goat anti-Rabbit 647**	Invitrogen	A21245		1:500	
**Goat anti-Mouse 448**	Invitrogen	A11029		1:500	
**Goat anti-Mouse 647**	Invitrogen	A21235		1:500	
**Chicken anti-Goat 488**	Invitrogen	A21467		1:600	
**Donkey anti-Rabbit 647**	Invitrogen	A31573		1:500	
